# Partial Rescue of F508del-CFTR Stability and Trafficking Defects by Double Corrector Treatment

**DOI:** 10.3390/ijms22105262

**Published:** 2021-05-17

**Authors:** Valeria Capurro, Valeria Tomati, Elvira Sondo, Mario Renda, Anna Borrelli, Cristina Pastorino, Daniela Guidone, Arianna Venturini, Alessandro Giraudo, Sine Mandrup Bertozzi, Ilaria Musante, Fabio Bertozzi, Tiziano Bandiera, Federico Zara, Luis J. V. Galietta, Nicoletta Pedemonte

**Affiliations:** 1 U.O.C. Genetica Medica, IRCCS Istituto Giannina Gaslini, 16147 Genova, Italy; valeriacapurro@yahoo.it (V.C.); valeriatomati@gaslini.org (V.T.); elvirasondo@gaslini.org (E.S.); cristinapastorino22@gmail.com (C.P.); ilaria.musante@unige.it (I.M.); federico.zara@unige.it (F.Z.); 2 Telethon Institute of Genetics and Medicine (TIGEM), 80078 Pozzuoli, Italy; m.renda@tigem.it (M.R.); a.borrelli@tigem.it (A.B.); d.guidone@tigem.it (D.G.); a.venturini@tigem.it (A.V.); 3 D3-PharmaChemistry, Fondazione Istituto Italiano di Tecnologia, 16163 Genova, Italy; alessandro.giraudo@iit.it (A.G.); fabio.bertozzi@iit.it (F.B.); tiziano.bandiera@iit.it (T.B.); 4 Analytical Chemistry Lab, Istituto Italiano di Tecnologia, Via Morego 30, 16163 Genova, Italy; sine.bertozzi@iit.it; 5 Department of Neurosciences, Rehabilitation, Ophthalmology, Genetics, Maternal and Child Health (DINOGMI), University of Genoa, 16132 Genova, Italy; 6 Department of Translational Medical Sciences (DISMET), University of Naples Federico II, 80131 Naples, Italy

**Keywords:** VX-445, elexacaftor, conformational stability, allosteric folding correction, chloride secretion, primary bronchial cells

## Abstract

Deletion of phenylalanine at position 508 (F508del) in the CFTR chloride channel is the most frequent mutation in cystic fibrosis (CF) patients. F508del impairs the stability and folding of the CFTR protein, thus resulting in mistrafficking and premature degradation. F508del-CFTR defects can be overcome with small molecules termed correctors. We investigated the efficacy and properties of VX-445, a newly developed corrector, which is one of the three active principles present in a drug (Trikafta^®^/Kaftrio^®^) recently approved for the treatment of CF patients with F508del mutation. We found that VX-445, particularly in combination with type I (VX-809, VX-661) and type II (corr-4a) correctors, elicits a large rescue of F508del-CFTR function. In particular, in primary bronchial epithelial cells of CF patients, the maximal rescue obtained with corrector combinations including VX-445 was close to 60–70% of CFTR function in non-CF cells. Despite this high efficacy, analysis of ubiquitylation, resistance to thermoaggregation, protein half-life, and subcellular localization revealed that corrector combinations did not fully normalize F508del-CFTR behavior. Our study indicates that it is still possible to further improve mutant CFTR rescue with the development of corrector combinations having maximal effects on mutant CFTR structural and functional properties.

## 1. Introduction

Cystic fibrosis (CF) is one of the most frequent genetic diseases among individuals of Caucasian origin, with nearly one child affected every 3500 births. CF is caused by mutations occurring in the CFTR gene encoding for the Cystic Fibrosis Transmembrane Conductance Regulator protein, a chloride-permeable channel expressed in epithelial cells of various organs, including the lungs, pancreas, intestine, liver, and sweat glands [1,2]. More than 2000 variants in the CFTR gene have been described, but to date, pathogenicity has been demonstrated only for 360 of them (CFTR2 database: https://cftr2.org (accessed on 1 September 2020)).

CF-associated mutations impair CFTR function by different mechanisms, including impaired cell surface expression due to mutant protein misfolding and premature degradation, or defective CFTR channel gating [3,4]. Some mutations, however, display a promiscuous mechanism, as in the case of F508del, the most frequent in CF patients, leading to both defective maturation and gating [3,4]. 

In the last decade, several drugs able to restore defective mutant CFTR activity have been approved for the treatment of CF patients [3,5]. In this respect, molecules that improve CFTR activity by increasing its open channel probability are called “potentiators”, whereas molecules that improve the proper folding and stability of the mutant protein, increasing its trafficking to the plasma membrane, are called “correctors” [3,5,6].

The various CFTR correctors are thought to work with various mechanisms of action so that combinations of correctors acting at different levels can lead to additive mutant CFTR rescue [7,8]. Indeed, it has been proposed that correctors exert their activity by interacting with different CFTR domains [8]. In this respect, CFTR has two nucleotide binding domains (NBD1 and NBD2), each one linked at the carboxy-terminus of a membrane-spanning domain (MSD1 and MSD2) [9,10]. A regulatory (R) domain connects the two MSD1-NBD1 and MSD2-NBD2 together [9,10]. Importantly, F508del does not only affect the stability of NBD1, where it is localized, but also affects other CFTR domains [9,10]. Small molecules that possibly stabilize interaction between mutant NDB1 and the membrane spanning domains (MSDs) are defined as type I correctors [8]. Molecules that potentially target NBD2 belong to type II correctors. Finally, small molecules that likely improve the folding and/or stability of NBD1 are classified as type III correctors. According to this nomenclature, VX-809 [11] and its structural analog VX-661 [12] (also known as lumacaftor and tezacaftor, respectively) are considered type I molecules [8], whereas VX-445 (elexacaftor; [13]) has been recently proposed to act with a type III mechanism of action [14]. Although the precise mechanism of action and binding site of correctors have not been conclusively determined, it is convenient to group correctors in separate classes based on functional evidence, i.e., the presence of additivity/synergy when compounds belonging to separate classes are combined together.

Presently, VX-661 and VX-445 are included, together with the potentiator VX-770 (ivacaftor), in a triple drug combination (Trikafta^®^/Kaftrio^®^) for the treatment of CF patients with one or two copies of F508del mutation. The triple combination, which elicits synergistic effects on CFTR protein maturation and function, appears to provide a significant clinical benefit with respect to previous treatments, consisting of either VX-809 plus VX-770 or VX-661 plus VX-770, without the second corrector [15]. In the present study, we analyzed the rescue of F508del-CFTR by VX-445 in combination with type I correctors to understand the extent of normalization with respect to wild type CFTR protein. Our results show that, despite a high level of functional rescue by the triple combination (two correctors plus a potentiator), F508del-CFTR protein is still characterized by a significant level of instability and degradation.

## 2. Results

**Figure 1:**
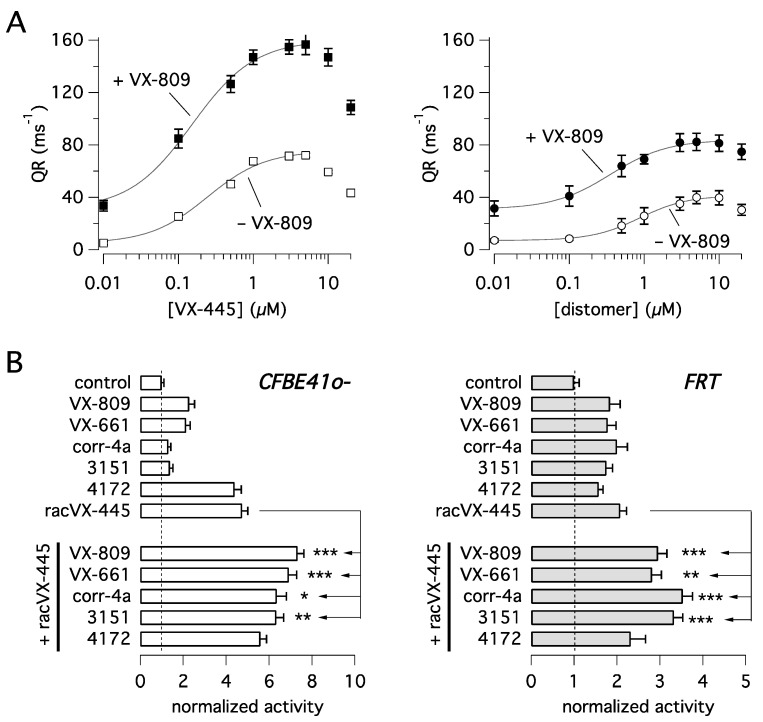
Functional evaluation of VX-445 corrector activity in heterologous expression systems. Compounds were tested on CFBE41o- and FRT cells expressing F508del-CFTR and the halide-sensitive yellow fluorescent protein (HS-YFP). (**A**) Dose-responses relationships for VX-445 and its *(R)*-enantiomer (i.e., distomer), as single agents or in the presence of VX-809 (1 µM) on CFBE41o- cells. (**B**) Evaluation of corrector combination. The graphs report F508del-CFTR activity in CFBE41o- (left) and FRT (right) cells treated with vehicle alone (DMSO) or VX-809 (3 µM), VX-661 (10 µM), corr-4a (5 µM), 3151 (10 µM), 4172 (10 µM), racVX-445 (3 µM), or their combinations. Symbols indicate statistical significance versus treatment with racVX-445 alone: *, *p* < 0.05; **, *p* < 0.01; ***, *p* < 0.001.

VX-445 carries a stereogenic center on the pyrrolidine moiety of its structure and therefore could exist as two stereoisomers. The compound approved for use is the enantiomer featuring the (*S*)-absolute configuration. Intrigued by the possible different stereospecificity elicited by the two enantiomers in rescuing mutant CFTR activity, we treated CFBE41o- cells expressing F508del-CFTR for 24 h with each of the two stereoisomers. We used compounds purchased from commercial sources as well as synthesized in-house [16]. After treatment, F508del-CFTR function was determined with the HS-YFP functional assay [17]. Figure 1A shows that VX-445 ((*S*)-enantiomer) acts as the eutomer showing significantly higher potency and efficacy relative to the opposite (*R*)-enantiomer, i.e., the distomer, whose activity is however not negligible. Co-treatment with VX-809 (1 µM) strongly amplified the rescue promoted by the two compounds. Interestingly, apparent affinity appeared to be affected by combination with VX-809, decreasing the EC_50_ of VX-445 from 254 nM when tested alone to 152 nM in the presence of VX-809. A similar behavior was also observed for the (*R*)-enantiomer, with EC_50_ decreasing from 852 nM to 354 nM. Dose-response relationships also revealed the presence of a dose-dependent inhibition of F508del-CFTR function at the highest VX-445 concentrations (> 5 µM). This phenomenon appears to restrict the concentration range producing maximal F508del-CFTR rescue. We also tested VX-445 as a racemate (racVX-445, i.e., the mixture of both (S)- and (*R*)-enantiomer) and compared its activity with that of other known CFTR correctors (Figure 1B). Such experiments were done in parallel on CFBE41o- and FRT cells, both with stable expression of F508del-CFTR and the HS-YFP. In CFBE41o- cells, racVX-445 and 4172, both classified as type III compounds [14,18], were largely more effective than the other correctors, belonging to different types/classes. In FRT cells, all compounds showed comparable activity. The most striking difference between the two cell models regarded corr-4a and 3151, compounds classified as type II correctors [8,19], which were poorly effective in CFBE41o- cells as single agents. 

We then investigated the possible corrector combinations (Figure 1B). In agreement with its functional classification as a type III corrector, racVX-445 generated significant additive/synergistic effects when combined with VX-809, VX-661, 3151, and corr-4a, but not with 4172. In particular, in CFBE41o- cells, corr-4a and 3151, which were nearly ineffective by themselves, markedly enhanced F508del-CFTR function when combined with rac-VX-445 (Figure 1B).

**Figure 2:**
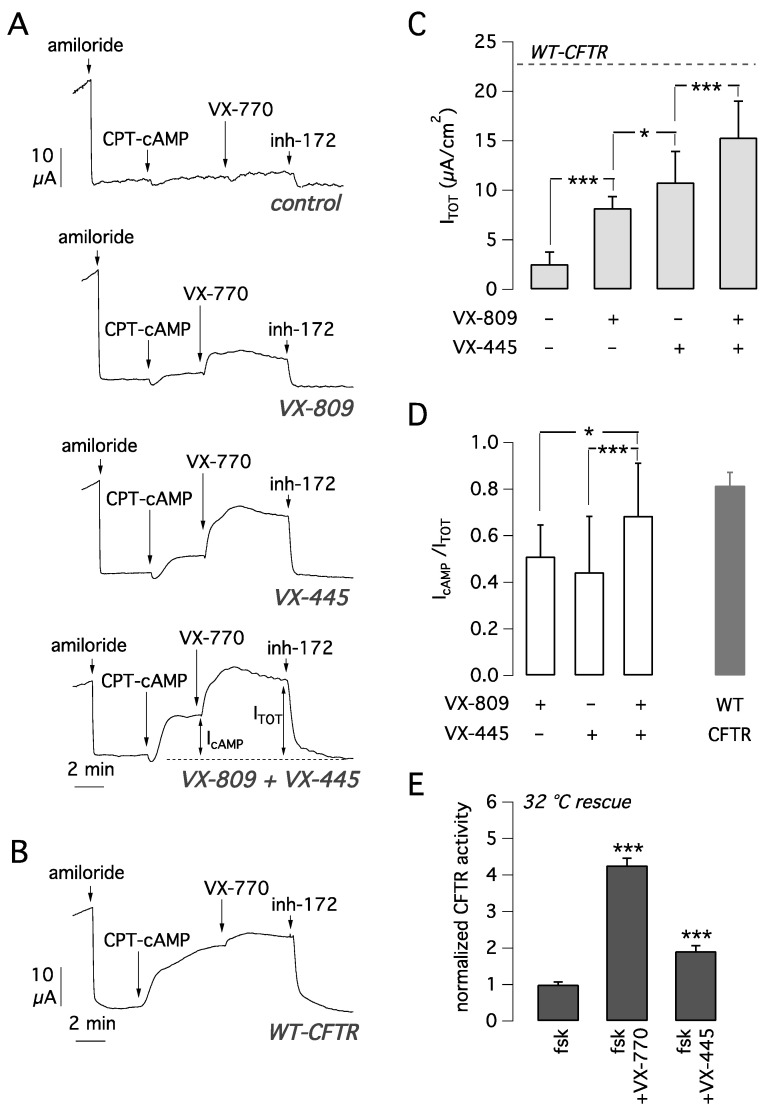
Functional evaluation of VX-445 corrector activity in human bronchial epithelial cells. (**A**) Effect of 24 h cell treatment with vehicle alone (DMSO), VX-445 (5 µM), VX-809 (1 µM), or both correctors together. Experiments were done on F508del/F508del bronchial epithelial cells derived from 5 different subjects (BE86, BE91, BE93, BE111, and BE115) with the short-circuit current technique. Representative recordings are from BE115. As parameters of CFTR function, we measured the total current sensitive to inh-172 (I_TOT_) and the current elicited by the cAMP analog alone (I_cAMP_). (**B**) Representative trace obtained from short-circuit current recordings on non-CF bronchial epithelial cells (BE99). (**C**) Summary of I_TOT_ (mean ± SD, *n* = 26–31) measured in F508del/F508del epithelia, derived from 5 different subjects (BE86, BE91, BE93, BE111, and BE115), exposed to indicated treatments. The dashed line reports the value of I_TOT_ in non-CF bronchial cells. *, *p* < 0.05; ***, *p* < 0.001 between indicated groups of data. (**D**) Ratio between the current elicited by cAMP stimulation (I_cAMP_) and total CFTR current (I_TOT_) in F508del/F508del epithelia treated with the indicated compounds (data obtained from experiments shown in **A** and **C**). *, *p* < 0.05; ***, *p* < 0.001 between indicated groups of data. (**E**) Evaluation of VX-445 as potentiator on F508del-CFTR CFBE41o- cells rescued at 32 °C for 24 h (mean ± SD, *n* = 5). ***, *p* < 0.001 vs. control.

The activity of VX-445 and VX-809, as single agents or in combination, as mutant CFTR correctors was analyzed on primary bronchial epithelial cells derived from F508del homozygous patients. To this aim, bronchial epithelia differentiated under air-liquid conditions were treated for 24 h by adding the compounds to the basolateral medium. The following day, the epithelia were mounted in a perfusion chamber to measure CFTR-dependent Cl^−^ secretion with the short-circuit current technique (Figure 2A). After inhibition of the epithelial sodium channel ENaC with amiloride (10 µM), cells were stimulated with the membrane permeable cAMP analogue CPT-cAMP (100 µM) followed by VX-770 (1 µM) to maximally activate F508del-CFTR. Then, CFTR currents were blocked adding the CFTR inhibitor-172 (inh-72, 10 µM) (Figure 2A). The amplitude of the current drop caused by inh-172 was taken as an estimate of total CFTR activity in each epithelium.

In epithelia treated for 24 h with vehicle alone (DMSO), total CFTR activity was relatively small (Figure 2A,C). Treatment with VX-809 or VX-445 caused a significant three-fold and four-fold increase in CFTR-mediated current, respectively, compared to vehicle-treated cells (Figure 2A,C). Treatment with the combination VX-445 plus VX-809 caused a six-fold current increase, an effect that was significantly higher than that of each corrector alone (Figure 2A,C). We also evaluated CFTR currents in non-CF cells with the same activating cocktail consisting of CPT-cAMP plus VX-770 (Figure 2B). The average CFTR current was 23.1 ± 3.5 µA (mean ± SD, *n* = 14). Interestingly, the total current activated in CF cells with the VX-809/VX-445 combination was 15.3 ± 3.7 µA (*n* = 26). Therefore, we can conclude that the rescue obtained with the double corrector combination is 66% of CFTR function in non-CF cells.

We also calculated the fraction of CFTR current elicited by CPT-cAMP alone with respect to the total current (I_cAMP_/I_TOT_). This parameter can be used to estimate the extent of the channel gating defect. A low value indicates that the cAMP stimulus is not enough to fully activate the channel, thus requiring a further stimulus represented by the potentiator. Whereas with either VX-809 or VX-445 alone I_cAMP_/I_TOT_ was 0.4–0.5 (Figure 2D), the corrector combination resulted in a significant increase in I_cAMP_/I_TOT_ up to ~0.7, close to the value calculated for non-CF cells (I_cAMP_/I_TOT_ = 0.8). This result suggests that treatment with the combination of the two correctors induces a change in F508del-CFTR protein conformation/stability that results in improved gating. 

Another possible reason is that VX-445 also acts as a potentiator, as suggested by others [20]. To address this possibility, we tested VX-445 (5 µM) acutely on CFBE41o- cells expressing F508del-CFTR that were previously kept for 24 h at 32 °C as a rescue maneuver. The results demonstrated that VX-445 behaves as a very weak potentiator (Figure 2E), the net effect being only 28% of the VX-770 activity (Figure 2E). 

**Figure 3:**
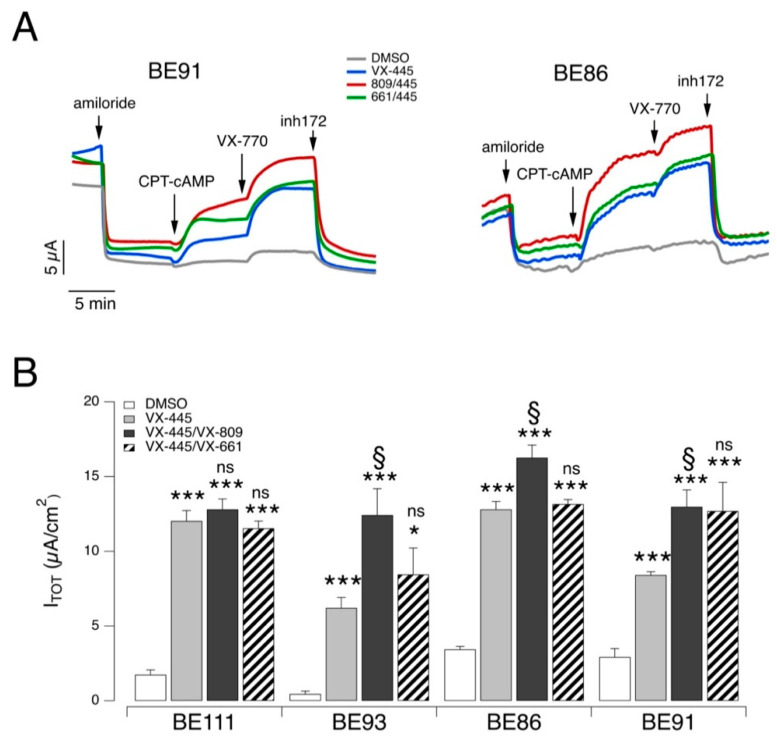
Functional evaluation of VX-445-based combinations on F508del/F508del human bronchial epithelial cells. (**A**) Representative traces of the effect of vehicle alone (DMSO) or VX-445 (3 µM), VX-809 (3 µM), or VX-661 (10 µM) as single agents or combinations in F508del/F508del bronchial epithelial cells (BE91 and BE86) with the short-circuit current technique. (**B**) Summary of results obtained from short-circuit current recordings on F508del/F508del bronchial epithelial cells derived from four different CF patients. Data reported are the amplitude of the current blocked by 10 µM inh-172 (I_TOT_; mean ± SD, *n* = 5). Asterisks indicate the statistical significance of single correctors vs. control (DMSO-treated): *, *p* < 0.05; ***, *p* < 0.001. Other symbols indicate the statistical significance of combinations of correctors vs. VX-445 alone: §, *p* < 0.05; ns, not significant.

Experiments on airway epithelial cells were also done by combining VX-445 with VX-661 since these two correctors are the ones included in the Trikafta^®^/Kaftrio^®^ medication [13]. The experiments were done on epithelia derived from four F508del homozygous patients. In this set of experiments, the efficacy of the VX-661/VX-445 combination was compared to that of VX-809/VX-445 and to VX-445 alone (Figure 3). The effect of the two correctors’ combinations was essentially similar on the different epithelia, although the combination VX-809/VX-445 was consistently the most effective. More precisely, in epithelia from three out of four patients (BE93, BE86, and BE91), the CFTR-mediated current with the VX809/VX445 combination was significantly larger than that of VX-445 alone. Instead, epithelia deriving from a fourth patient (BE111) showed a high response to VX-445 so that the rescue with this single agent was comparable to that obtained with the double corrector combinations (Figure 3B). 

**Figure 4:**
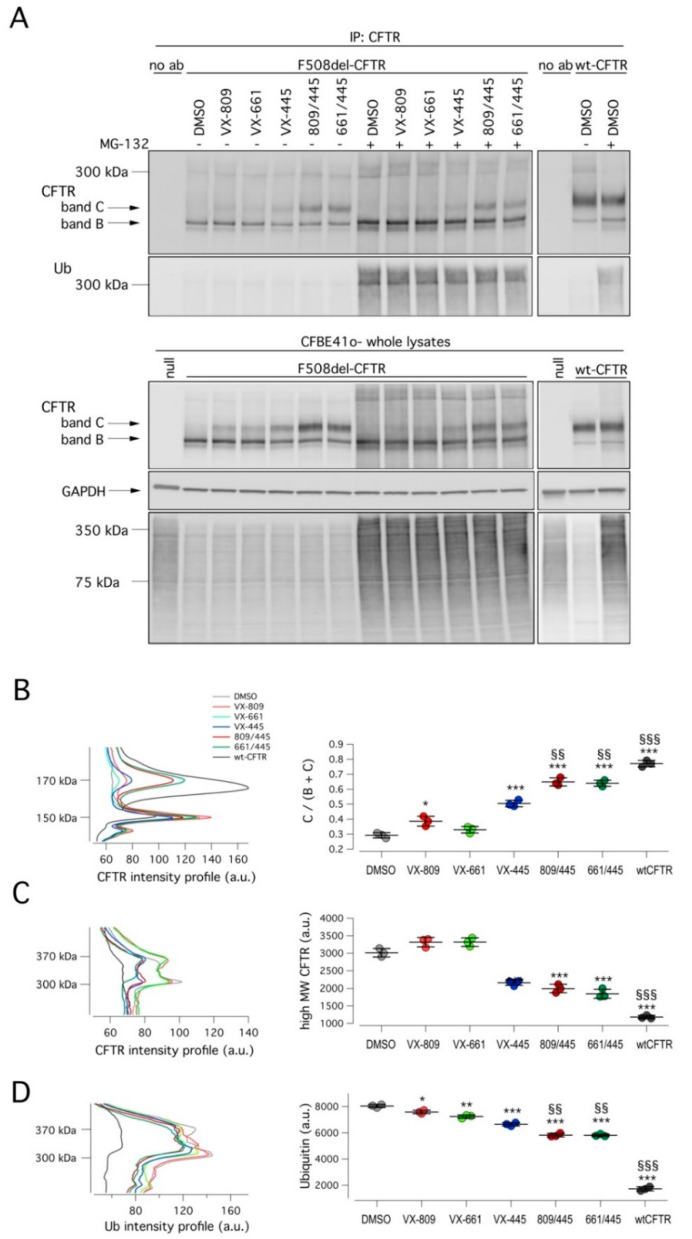
Effect of VX-445-based treatments on mutant CFTR ubiquitylation. (**A**) Biochemical analysis of CFTR ubiquitylation and expression pattern in CFTR immunoprecipitates from wild-type or F508del-CFTR expressing CFBE41o- cells after 24 h treatment with vehicle alone (DMSO), or (for mutant CFTR only) VX-445 (3 µM), VX-809 (3 µM), or VX-661 (10 µM) as single agents or combinations in the absence or in the presence of the proteasome inhibitor MG-132 (10 µM; last 4 hr) to block proteasomal degradation. For comparison, whole lysates derived from CFBE41o- cells not expressing CFTR (null cells) are also shown as controls for antibody specificity. (**B**–**D**) Representative density profiles (left graphs) and corresponding quantification (right graphs) of CFTR and ubiquitin in the absence (**B**) or in the presence of MG-132 (**C**,**D**) Quantification of the density profiles was performed by integrating the profile curves in the selected intervals of molecular weight (see left graphs). *, *p* < 0.05; **, *p* < 0.01; ***, *p* < 0.001 vs. DMSO; §§, *p* < 0.01; §§§, *p* < 0.001 vs. VX-445 alone.

To further characterize the corrector activity of VX-445, alone or combined with a type I corrector, we evaluated mutant CFTR expression and ubiquitylation status. To this aim, cells were treated for 24 h with DMSO (vehicle), VX-809, VX-661, VX-445, or their combinations. Subsequently, degradation through the proteasomal pathway was blocked by treating cells for 4 h with a proteasome inhibitor MG-132 (10 µM) or vehicle, and then cells were lysed. Cell lysates were immunoprecipitated using an anti-CFTR antibody and then subjected to SDS-PAGE followed by Western blotting to evaluate CFTR maturation and ubiquitylation (Figure 4). Treatment with correctors rescued mutant CFTR, as shown by the appearance of the mature, fully glycosylated form of CFTR (band C) in total cell lysates and in immunoprecipitates (Figure 4). In particular, the largest increase of band C intensity was obtained with the corrector combinations in agreement with functional data (Figure 4A,B). As expected, the block of proteasomal degradation by MG-132 caused the appearance of high molecular weight CFTR forms (at 300–350 kDa), corresponding to polyubiquitinated CFTR forms. As demonstrated by immunoprecipitation experiments, the MG-132 effect was caused by the accumulation of ubiquitylated CFTR. Interestingly, the treatment with correctors decreased but did not abolish ubiquitylation (Figure 4A,C). F508del-CFTR still showed a significant degree of ubiquitylation and hence degradation even in the presence of VX-445/VX-809 or VX-445/VX-661 treatments.

**Figure 5:**
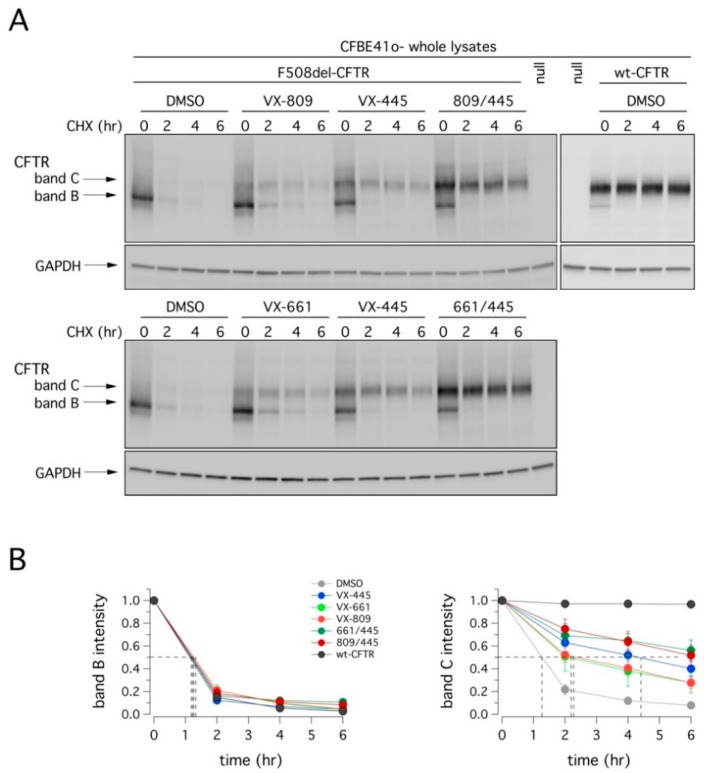
Effect of VX-445-based treatments on mutant CFTR half-life. (**A**) Immunoblot detection of CFTR in whole lysates derived from wild-type or F508del-CFTR expressing CFBE41o- cells treated with vehicle alone (DMSO), or (for mutant CFTR only) with VX-445 (3 µM), VX-809 (3 µM), or VX-661 (10 µM) as single agents or combinations, at different time points following CHX-induced block of protein synthesis. For comparison, whole lysates derived from CFBE41o- cells not expressing CFTR (null cells) are also shown as controls for antibody specificity. (**B**) Quantification of wild-type or mutant CFTR (band B and band C) half-life in experiments detailed in (**A**), normalized by the value at time = 0. Data are means ± SD (*n* = 3). Dashed lines indicate 50% of the protein remaining (*y*-axis) and the corresponding intercepts on the *x*-axis, indicating the estimated half-life.

We also evaluated the degradation rate of F508del-CFTR in CFBE41o- cells after 24 h incubation with VX-445, VX-809, or VX-661 as single agents or as combinations, followed by treatment with cycloheximide (CHX) to block protein synthesis. Cells were then lysed at different time points, and cell lysates were subjected to SDS-PAGE followed by immunoblotting to evaluate CFTR expression. As shown in Figure 5 and Table 1, the expression of mutant CFTR (both band B and C) decreased over time. Treatment with VX-809 or VX-661 significantly increased the half-life of mature CFTR (band C) by two-fold. The effect of VX-445 was a four-fold increase. The combinations VX-445/VX-809 or VX-445/VX-661 were equally effective and producing the largest effect on F508del-CFTR stability, with a half-life of nearly 6 h. Although this value represents a significant improvement compared to vehicle-treated cells (~1 h), it was considerably lower than that of wild type CFTR (exceeding 12 h; [21,22]). 

**Figure 6:**
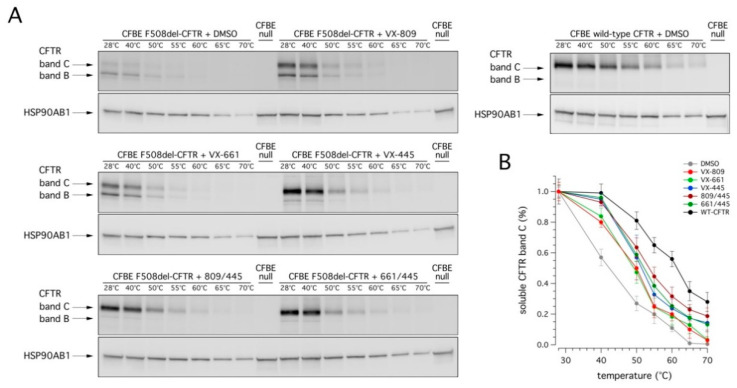
Effect of VX-445-based treatments on the conformational stability of mutant CFTR. (**A**) Immunoblot detection of CFTR in whole lysates derived from wild-type or F508del-CFTR expressing CFBE41o- cells treated with vehicle alone (DMSO), or (for mutant CFTR only) with VX-445 (3 µM), VX-809 (3 µM), or VX-661 (10 µM) as single agents or combinations, following heat-denaturation at 28–70 °C. For comparison, whole lysates derived from parental CFBE41o- cells (null cells) are also shown as controls for antibody specificity. (**B**) Quantification of aggregation-resistant (soluble) CFTR band C by densitometry, normalized by HSP90AB1 expression (means ± SD; *n* = 3).

Finally, we evaluated the conformational stability of mutant CFTR protein under resting conditions and following rescue with correctors by determining the denaturation temperature, which induces the conversion of detergent solubilized CFTR into SDS-resistant aggregates [23,24]. Accordingly, F508del-CFTR CFBE41o- cells were treated for 24 h with VX-445, VX-809, or VX-661 as single agents or as combinations, and then lysed. Cell lysates were generated, in parallel, from wild type CFTR-expressing CFBE41o- cells. Cell lysates were heat-denatured at 28–70 °C and the aggregation-resistant CFTR protein was quantified by SDS-PAGE followed by Western blotting (Figure 6). 

Treatment with correctors significantly increased the resistance to thermoaggregation of mature CFTR (Figure 6). This effect was, however, modest following treatment with VX-809 or VX-661: the temperature at which 50% of the mature protein was still present increased from 42 °C (for DMSO) to nearly 50 °C (for either VX-809 or VX-661; see Figure 6B). On the contrary, in the presence of VX-445, the conformational stabilization of complex-glycosylated F508del-CFTR was more evident (reaching nearly 52 °C; see Figure 6B). No significant difference was detected following combined treatment with VX-445 and VX-661; however, a modest improvement was observed when VX-445 was combined with VX-809 (54 °C; see Figure 6B). Also in this case, despite the significant improvement following treatment with the VX-445-based combinations, the conformational stability of rescued F508del-CFTR was still markedly lower than that of wild type CFTR (Figure 6B). Indeed, the thermoaggregation curve obtained with the most effective treatment (VX-809/VX-445) was still different, by nearly 8 °C, with respect to that of wild type CFTR (reaching nearly 62 °C; see Figure 6B).

**Figure 7:**
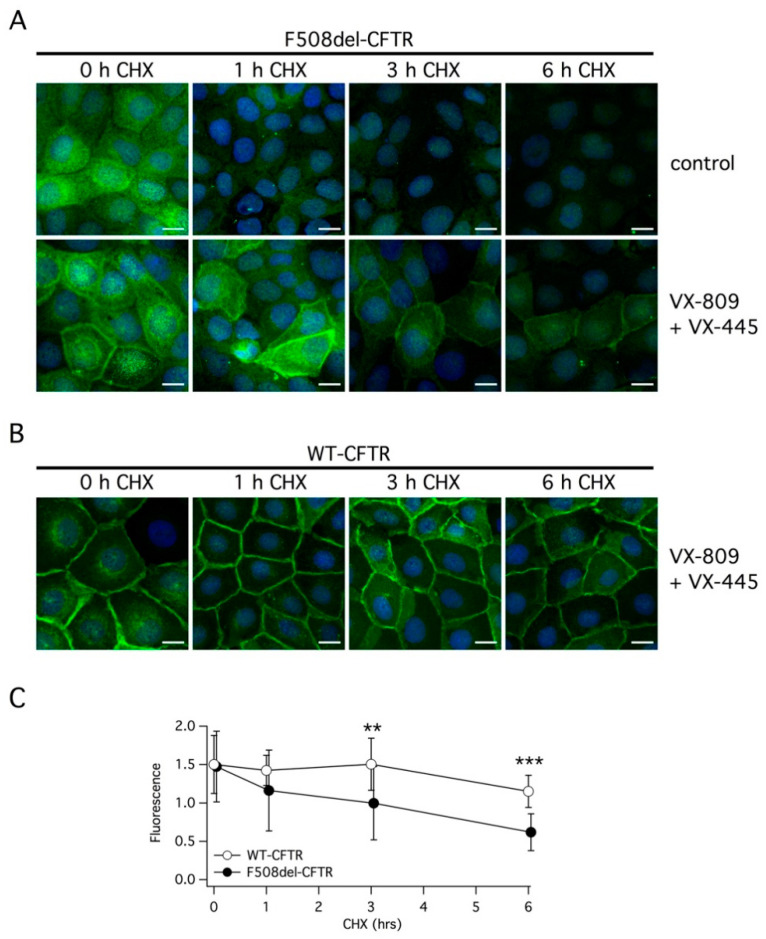
Analysis of CFTR subcellular localization. (**A**,**B**) Representative images showing the detection of F508del-CFTR or wild type CFTR protein in CFBE41o- cells by immunofluorescence. Cells were incubated with vehicle (DMSO) or with the corrector combination (5 µM VX-445 plus 1 µM VX-809) for 24 h. Cells were immediately fixed or treated for the indicated time (1–6 h) with CHX and then fixed (scale bar: 15 µm). (**C**) Analysis of CFTR protein expression in the plasma membrane at different times following CHX addition. **, *p* < 0.01; ***, *p* < 0.001.

We also investigated the effect of double corrector treatment on the subcellular localization of F508del-CFTR using immunofluorescence. Without treatment, the mutant protein was exclusively present in intracellular compartments (Figure 7A). The treatment with VX-445 plus VX-809 clearly caused the appearance of a peripheral signal, consistent with trafficking of the protein to the plasma membrane. However, under this condition, there was still a considerable amount of the protein remaining inside the cells compared to cells expressing wild type CFTR, in which the peripheral signal was largely prevalent (Figure 7B). Following the block of protein synthesis with CHX, we observed a very rapid rundown of F508del-CFTR. In cells treated with vehicle alone, the immunofluorescence signal was nearly cancelled by the 6 h treatment with CHX. In cells corrected with VX-445/VX-809, a rapid and marked decrease in F508del-CFTR expression was also observed (Figure 7A). In cells expressing wild type CFTR, the rundown of the protein following protein synthesis block was slower (Figure 7B). A significant difference between cells expressing wild type and mutant CFTR was detected at 3 and 6 h of CHX treatment (Figure 7C).

## 3. Discussion

The development of VX-445 has radically changed the therapeutic perspective for many patients with CF. Indeed, the inclusion of VX-445 (elexacaftor) with the corrector VX-661 (tezacaftor) and the potentiator VX-770 (ivacaftor) in a combinatorial drug therapy has demonstrated considerable clinical benefit even for patients with a single F508del allele [13,15,25,26,27]. This marked therapeutic effect is expected from the synergy of the two correctors that act with complementary mechanisms of action. VX-661 belongs to type I correctors, which are believed to support the formation of NBD1-MSD1 and NBD1-MSD2 interfaces, possibly by acting directly on the first transmembrane domain of CFTR [28,29] or by binding to NBD1 to improve the interaction between NBD1 and the intracellular loops [30,31]. VX-445 is instead a type III corrector [14] that is postulated to interact with and stabilize NBD1. The combined activity of the two correctors should overcome the multiple defects that F508del causes to CFTR folding and stability. Interestingly, when we examined the correction efficacy of single compounds and their combination in more detail, we observed that a large part of the effect, particularly with some assays and cell models, was due to VX-445 alone, with the added value of the second corrector being variable. In agreement with recently published results, we found that VX-445 behaves as a type III corrector since it shows additivity with VX-809, VX-661, corr-4a, and 3151 but not with 4172. In this regard, although corr-4a has been categorized as a type II corrector, there are lines of evidence suggesting that corr-4a acts as a proteostasis regulator rather than as a pharmacological chaperone. First, the activity of corr-4a is markedly cell type-dependent, thus influenced by cell background [7] as also shown in the present study. Second, corr-4a rescues misfolding defects of mutant proteins other than CFTR [32,33,34]. The additivity/synergy of VX-445 with type I correctors was found in functional assays, done in heterologous expression systems and primary airway epithelial cells, and in biochemical experiments. In particular, the combination of VX-445 with either VX-809 or VX-661 was particularly effective in the primary bronchial epithelial cells of CF patients. The net rescue effect was equivalent to 50–70% of CFTR-dependent Cl^-^ secretion in non-CF cells, a notable result that explains the clinical benefit of Trikafta^®^ compared to Orkambi^®^ [15,25,27]. However, despite this large effect, our experiments revealed that the combination of the two correctors does not fully prevent the folding and stability defects caused by F508del mutation.

First, when we analyzed the CFTR ubiquitylation status, we observed that treatments with VX-809 or VX-661 resulted in a very modest decrease of ubiquitylated CFTR, paralleled by a slight increase of high-molecular weight CFTR forms. Treatment with VX-445 was more effective, resulting in a decrease of ubiquitylated CFTR that was paralleled by a marked reduction of high-molecular weight CFTR forms. Finally, when cells were treated with either VX-809/VX-445 or VX-661/VX-445, a further decrease in both ubiquitylated and high-molecular weight CFTR forms was observed. However, despite the presence of the double corrector combination, a substantial amount of mutant CFTR was still subjected to ubiquitylation and degradation. 

Second, the analysis of CFTR degradation rate demonstrated that VX-445 significantly prolongs the half-life of mature CFTR (band C) 4-fold. Combination of VX-445 with either VX-809 or VX-661 further increased this value 5-fold, thus extending mutant F508del-CFTR half-life from approximately 1.25 h to approximately 6 h. To the best of our knowledge, such a result has only been reported before, under similar experimental conditions, for the triple combination 4172/VX-809/3151, i.e., by using three correctors having different mechanisms, 4172 and 3151 being a type III and a type II corrector, respectively [18]. Despite the relevance of such an increase of mutant CFTR half-life, this value is still far from reaching the estimated half-life of wild-type CFTR protein, exceeding 12 h [21,22]. 

Third, the analysis of protein conformational stability with the thermoaggregation assay confirmed that VX-445/VX-809 or VX-445/VX-661 did not fully restore the stability of F508del-CFTR to wild type protein levels. 

Finally, we found by immunofluorescence that the double corrector combination does not fully restore the trafficking of mutant CFTR to plasma membrane, since a significant amount of the protein remains in intracellular compartments. Upon protein synthesis block, F508del-CFTR rapidly disappears even in the presence of the correctors.

In addition to the trafficking problem, F508del-CFTR channel is also characterized by defective gating. In this regard, another interesting feature displayed by the VX-445-based treatments is the ability to, at least partially, overcome the gating defect caused by F508del. Indeed, in our experiments on bronchial epithelial cells, the fraction of F508del-CFTR activity that can be activated by cAMP, without the potentiator, was increased by the pretreatment with the combination of VX-445 plus VX-809. It has been proposed that VX-445 also has a potentiator activity [20]. However, when we tested VX-445 in a potentiator assay (acute application of VX-445 after rescue of F508del-CFTR at low temperature), we found only a partial activity, equivalent to 30% of VX-770. A possible explanation is that the effect of VX-445 on gating is not that of a classical CFTR potentiator such as VX-770. By cryo-EM, the VX-770 site of action was found in the transmembrane domains [35]. The binding site of VX-445 is still unknown. It is possible that stabilization of the mutant CFTR structure by binding to a site different from that of VX-770 has a beneficial effect on the conformational changes underlying channel gating.

Summarizing, the combination of correctors belonging to type I and III does not entirely overcome the molecular defects caused by F508del mutation. This may be due to a suboptimal efficacy of either VX-809 or VX-661. In fact, many of our experiments, including functional assays on primary airway epithelial cells, show that most mutant CFTR rescue achieved with the combination can be attributed to the effect of VX-445 alone. In particular, despite some interindividual variability, it appears that, on primary bronchial epithelia, the rescue by treatment with VX-445/VX-661 was never significantly different from that obtained with VX-445 alone. This partial efficacy of the present corrector combinations in normalizing mutant CFTR properties may not be a problem since a rescue of 50–70% of wild type CFTR function resembles, in theory, the condition of F508del carriers, who are notoriously devoid of symptoms. However, the development of even more active corrector combinations, possibly based on more effective type I correctors, may be desirable in the future, for example, to treat patients bearing mutations with an even more severe trafficking defect than that caused by the F508del mutation.

## 4. Materials and Methods

### 4.1. Chemicals

VX-445 (chemical name: *N*-(1,3-dimethylpyrazol-4-yl)sulfonyl-6-[3-(3,3,3-trifluoro-2,2-dimethylpropoxy)pyrazol-1-yl]-2-[(*S*)-2,2,4-trimethylpyrrolidin-1-yl]pyridine-3-carboxamide), tested in these studies as pure (*S*)-enantiomer, was either purchased from MedChemExpress (Monmouth Junction, NJ, USA) or synthesized in house [16].

racVX-445 as well as enantiomerically pure (>99% e.e.) (*R*)-enantiomer [*N*-(1,3-dimethylpyrazol-4-yl)sulfonyl-6-[3-(3,3,3-trifluoro-2,2-dimethylpropoxy)pyrazol-1-yl]-2-[(*R*)-2,2,4-trimethylpyrrolidin-1-yl]pyridine-3-carboxamide], tested in these studies, were either purchased from MedChemExpress (Monmouth Junction, NJ, USA) or synthesized in house (see Supporting Information for Chemistry: Synthetic Materials and Methods). 

VX-809, VX-661, and VX-770 were purchased from Selleck Chemicals LLC (Houston, TX, USA).

### 4.2. Cell Culture

FRT and CFBE41o^-^ cells stably expressing the halide-sensitive yellow fluorescent protein (HS-YFP) YFP-H148Q/I152L and F508del-CFTR were generated as previously described (see [36] for FRT cells; [37] for CFBE41o- cells). FRT cells were grown in Coon’s F-12 modified medium while CFBE41o- required MEM, in both cases supplemented with 10% fetal calf serum, 2 mM L-glutamine, 100 U/mL penicillin, and 100 µg/mL streptomycin. For the YFP-based assays of CFTR activity, FRT or CFBE41o^−^ cells were plated (100,000 cell/well or 50,000 cells/well, respectively) on clear-bottom 96-well black microplates (Corning Life Sciences, Glendale, AZ, USA). 

The methods for the isolation, culture, and differentiation of primary bronchial epithelial cells were previously detailed [38]. In brief, epithelial cells were obtained from mainstem human bronchi, derived from CF and non-CF individuals undergoing lung transplant. For the present study, cells were obtained from five F508del homozygous CF patients (BE86, BE91, BE93, BE111, and BE115) and one non-CF subject (BE99). Epithelial cells (detached by overnight treatment of bronchi with protease XIV) were cultured in a serum-free medium (LHC9 mixed with RPMI 1640, 1:1) containing various hormones and supplements, and favoring cell number expansion. For cells deriving from CF patients, the culture medium contained in the first days a complex mixture of antibiotics (usually colistin, piperacillin, and tazobactam) to eradicate bacteria. The collection of bronchial epithelial cells (supported by Fondazione per la Ricerca sulla Fibrosi Cistica through the “Servizio Colture Primarie”) and their study to investigate the mechanisms of transepithelial ion transport were specifically approved (on 8 July 2018) by the Ethics Committee of the Istituto Giannina Gaslini following the guidelines of the Italian Ministry of Health (registration number: ANTECER, 042-09/07/2018). Each patient provided informed consent to the study using a form that was also approved by the Ethics Committee. 

To obtain differentiated epithelia, cells were seeded at high density on porous membranes (Snapwell inserts, Corning, code 3801). After 24 h, the serum-free medium was removed from both sides and, on the basolateral side only, replaced with Pneumacult ALI medium (StemCell Technologies, Vancouver, BC, Canada), and the differentiation of cells (up to 16–18 days) was performed in air-liquid interface (ALI) condition. 

### 4.3. YFP-Based Assay for CFTR Activity

The HS-YFP microfluorimetric assay for the determination of CFTR activity was described in detail in previous studies [17,19,37,39]. Briefly, prior to the assay, cells were washed and then incubated for 25 min with 60 µL of PBS plus forskolin (20 µM) and VX-770 (1 µM) to maximally stimulate F508del-CFTR. Cells were then transferred to a microplate reader (FluoStar Galaxy or Fluostar Optima; BMG Labtech, Offenburg, Germany) for CFTR activity determination. The plate reader was equipped with high-quality excitation (HQ500/20X: 500 ± 10 nm) and emission (HQ535/30M: 535 ± 15 nm) filters for YFP (Chroma Technology, Olching, Germany). YFP fluorescence was recorded for 2 s before and 12 s after injection of 165 µL of an iodide-containing solution (PBS with Cl^−^ replaced by I^−^; final I^−^ concentration 100 mM). Data were normalized to the initial background-subtracted fluorescence. To determine I^-^ influx rate, the final 11 s of the data for each well were fitted with an exponential function to extrapolate initial slope (dF/dt).

### 4.4. Antibodies

The following antibodies were used: mouse monoclonal anti-CFTR (ab570 and ab596, J.R. Riordan, University of North Carolina at Chapel Hill, and Cystic Fibrosis Foundation Therapeutics); rabbit polyclonal anti-CFTR (ACL-006; Alomone Labs, Jerusalem, Israel; RRID:AB_2039804); mouse monoclonal anti-GAPDH (sc-32233; Santa Cruz Biotechnology, Dallas, TX, USA, Inc; RRID:AB_627679); mouse monoclonal anti-Ub (sc-8017; Santa Cruz Biotechnology, Dallas, TX, USA, Inc; RRID:AB_2762364); rabbit polyclonal anti-HSP90AB1 (SAB4300541; Sigma-Aldrich, St. Louis, MO, USA; RRID:AB_10629523); horseradish peroxidase (HRP)-conjugated anti-mouse IgG (ab97023; Abcam, Cambridge, UK; RRID:AB_10679675); or HRP-conjugated anti-rabbit IgG (31460; Thermo Fisher Scientific, Waltham, MA, USA; RRID:AB_228341).

### 4.5. CFTR Immunoprecipitation (IP) Assay

Parental CFBE41o- cells (null cells, lacking CFTR expression) or CFBE41o- cells stably expressing either wild-type or mutant F508del-CFTR were grown to confluence on 60-mm diameter dishes and treated for 24 h with the indicated compounds in the absence or in the presence of MG-132 (10 µM) in the last 4 h. Then, cells were rinsed twice with ice-cold PBS without Ca^2+^/Mg^2+^ and then lysed with IP Lysis Buffer (#87788, Thermo Fisher Scientific, Waltham, MA, USA) containing EDTA-free complete protease inhibitor (Roche Molecular Systems, Inc, Basilea, Swiss), N-ethylmaleimide (5 mM) and MG-132 (20 µM). Nuclei were pelleted by centrifugation at 15,000× *g* at 4 °C for 20 min. Supernatant protein concentration was calculated using the BCA assay (Euroclone, Pero (MI), Italy) following the manufacturer’s instructions. An aliquot of supernatant corresponding to 600 μg of protein was incubated for 1 h with 2 μg/sample of rabbit polyclonal anti-CFTR antibody (Alomone Labs, Jerusalem, Israel), with rocking at room temperature (RT). An antibody-antigen mixture was precipitated with 25 μL/sample of Pierce Protein A/G Magnetic Beads (Thermo Fisher Scientific, Waltham, MA, USA) for 1 h rocking at RT, following supplier’s instructions. Immunoprecipitated proteins were eluted from the resin under reducing conditions with 100 μL Laemli Sample Buffer 1X, at RT. Equal amounts of IP products were analyzed by Western blotting (20 μL).

### 4.6. Western Blot

CFBE41o- cells were grown to confluence on 60-mm diameter dishes and lysed in RIPA buffer (50 mM Tris-HCl pH 7.4, 150 mM NaCl, 1% Triton X-100, 0.5% Sodium deoxycholate, 0.1% SDS) containing a complete protease inhibitor (Roche). Cell lysates were then processed as previously described [40]. In brief, after centrifugation at 15,000× *g* at 4 °C for 10 min, supernatant protein concentration was calculated using the BCA assay (Euroclone, Pero (MI), Italy) following the manufacturer’s instructions. Equal amounts of protein (10 μg to detect CFTR and GAPDH) were separated onto gradient (4–15%) Criterion TGX Precast gels (Bio-rad laboratories Inc., Hercules, CA, Stati Uniti), transferred to a nitrocellulose membrane with Trans-Blot Turbo system (Bio-rad Laboratories Inc., Hercules, CA, Stati Uniti), and analyzed by Western blotting. Proteins were detected using antibodies indicated in the dedicated methods section and subsequently visualized by chemiluminescence using the SuperSignal West Femto Substrate (Thermo Fisher Scientific, Waltham, MA, USA). Chemiluminescence was monitored using the Molecular Imager ChemiDoc XRS System. Images were analyzed with ImageJ software (National Institutes of Health). Bands were analyzed as a region of interest (ROI), normalized against the GAPDH loading control. The molecular weight of the proteins (on the basis of the Precision Plus Protein WesternC Standards, Bio-rad Laboratories Inc., Hercules, CA, Stati Uniti) and the lane density profiles of ubiquitylated CFTR were calculated using the band analysis program of the software Quantity one 4.6 of the Molecular Imager ChemiDoc XRS System. Quantification of the density profiles was performed by calculating the area under the profile curves in selected intervals of molecular weight. Data are presented as mean ± SEM of independent experiments. 

To evaluate CFTR half-life, CFBE41o- cells were treated with cycloheximide (CHX; 150 µg/mL) (SigmaAldrich, St. Louis, MO, USA) 24 h after treatment with test compounds. At different time points (0, 2, 4, 6 h), cells were then lysed in RIPA buffer 1X and subjected to SDS-PAGE, as previously described [41].

Uncropped Western Blot images are shown in Supplementary Information: Uncropped WB Images.

### 4.7. Thermoaggregation Assay

Thermoaggregation assays were performed, as previously described [17,24]. In brief, 24 h after treatment with test compounds, CFBE41o- cells were treated for 2 h with CHX (150 µg/mL) and then lysed in RIPA buffer on ice. Lysates were cleared by centrifugation at 15,000× *g* for 10 min at 4 °C. Equal amounts of lysates (50 µg) were exposed to different temperatures (28, 40, 50, 55, 60, 65, 70 °C) for 15 min using polymerase chain reaction thermocyclers (Finnzymes-Thermo Fisher Scientific, Waltham, MA, USA). Macromolecular aggregates were eliminated by centrifugation at 15,000× *g* for 15 min at 4 °C. The remaining soluble wild-type and F508del-CFTR, HSP90AB1 (as loading control), in the supernatant were measured by quantitative immunoblotting to evaluate the aggregation tendency.

### 4.8. Immunofluorescence

CFBE41o- cells expressing F508del- or wild-type CFTR were seeded on µ-Slide 12 well removable chamber support (Ibidi, Gräfelfing, Germany) at a density of 20,000 cells per well in a total volume of 200 µL of complete medium. After 24 h, cells were treated with the corrector combination (1 µM VX-809, 5 µM VX-445) or with vehicle (DMSO). After a further 24 h, cells were either immediately fixed by adding 200 µL of 10% neutral buffered formalin (0501005Q, Bio-Optica, Milan, Italy) or treated for 1–6 h with 100 µg/mL cycloheximide to inhibit protein synthesis and then fixed. Fixed cells were washed three times in PBS and then permeabilized with 0.05% saponin in blocking buffer (0.5% BSA, 50 mM NH_4_Cl, 0.02% NaN_3_) for 30 min at room temperature. Cells were incubated overnight at 4 °C with 200 μL of primary antibody diluted in the blocking buffer. Mouse IgG1 anti-CFTR ab570 at 1:200 was used as the primary antibody.

Following incubation with primary antibody, cells were rinsed three times in PBS and then incubated with 200 μL of a solution of secondary Alexa Fluor 488 conjugated antibody (Invitrogen) diluted 1:200 in blocking buffer for 1 h in the dark. After three further washes in PBS, the silicone chamber was removed and cells were mounted with Fluoroshield with DAPI (Millipore-SigmaAldrich, St. Louis, MO, USA) to stain cell nuclei. Image acquisition was performed using a laser scanning confocal microscope (TCS SPE; Leica Microsystems, Wetzlar, Germany). Image analysis was performed using Leica and ImageJ (NIH) software. A region of interest (ROI) was used to measure the average fluorescence intensity in the plasma membrane. For each cell, four ROIs were considered and the results averaged. Forty cells per condition were analyzed.

### 4.9. Short-Circuit Current Recordings

Snapwell inserts carrying differentiated bronchial epithelia were mounted in a vertical diffusion chamber resembling a Ussing chamber with internal fluid circulation. Both apical and basolateral hemichambers were filled with 5 mL of a solution containing (in mM): 126 NaCl, 0.38 KH_2_PO_4_, 2.13 K_2_HPO_4_, 1 MgSO_4_, 1 CaCl_2_, 24 NaHCO_3_, and 10 glucose. Both sides were continuously bubbled with a gas mixture containing 5% CO_2_–95% air and the temperature of the solution was kept at 37 °C. The transepithelial voltage was short-circuited with a voltage clamp (DVC-1000, World Precision Instruments; VCC MC8 Physiologic Instruments) connected to the apical and basolateral chambers via Ag/AgCl electrodes and agar bridges (1 M KCl in 1% agar). The offset between voltage electrodes and the fluid resistance were adjusted to compensate parameters before experiments. The short-circuit current was recorded by analogical to digital conversion on a personal computer.

### 4.10. Statistics 

The Kolmogorov–Smirnov test was used to assess the assumption of normality of data distribution. An analysis of variance (ANOVA) followed by a post-hoc test was used when comparing more than two groups in order to avoid multiple comparisons error. For normally distributed quantitative variables, a parametric ANOVA was performed. 

The statistical significance of the effect of single drug treatments on CFTR activity in FRT, CFBE41o-, or HBE cells was tested by parametric ANOVA followed by the Dunnet multiple comparisons test (all groups against the control group) as a post hoc test. In the case of combinations of drugs, statistical significance was verified by ANOVA followed by the Tukey test (for multiple comparisons) as a post hoc test. When comparing selected pairs of treatment, the statistical significance was tested by ANOVA followed by Bonferroni as a post hoc test.

Normally distributed data are expressed as mean ± SD and significances are two-sided. Differences were considered statistically significant when *p* < 0.05.

## Figures and Tables

**Table 1:** Estimated half-life of mutant CFTR following rescue by single or double corrector treatment.

		T = 2 h			T = 4 h			T = 6 h		
	Band C Half-Life	DMSO	809/445	661/445	DMSO	809/445	661/445	DMSO	809/445	661/445
DMSO	1.25 h									
VX-809	2.25 h	**			**			*		
VX-661	2.25 h	*			*			*		
VX-445	4.5 h	***	ns	ns	***	ns	ns	***	ns	ns
809/445	6 h	***			***			***		
661/445	6 h	***			***			***		
wt-CFTR	>6 h	***	ns	*	***	**	**	***	***	***

For each time-point, symbols indicate statistical significance vs. the indicated condition: *, *p* < 0.05; **, *p* < 0.01; ***, *p* < 0.001; ns = not significant.

## Data Availability

The data presented in this study are available in the article and supplementary material.

## Supplementary Materials

The following are available online at https://www.mdpi.com/article/10.3390/ijms22105262/s1, Supporting Information for Chemistry: Synthetic Materials and Methods; Supplementary Information: Uncropped WB Images.
