# Supplementary material for: Partial Rescue of F508del-CFTR Stability and Trafficking Defects by Double Corrector Treatment
Source: Int J Mol Sci. 2021 May 17;22(10):5262. doi: 10.3390/ijms22105262 (PMC8156943; doi:10.3390/ijms22105262)

Fig 4A **IP:CFTR – WB CFTR**

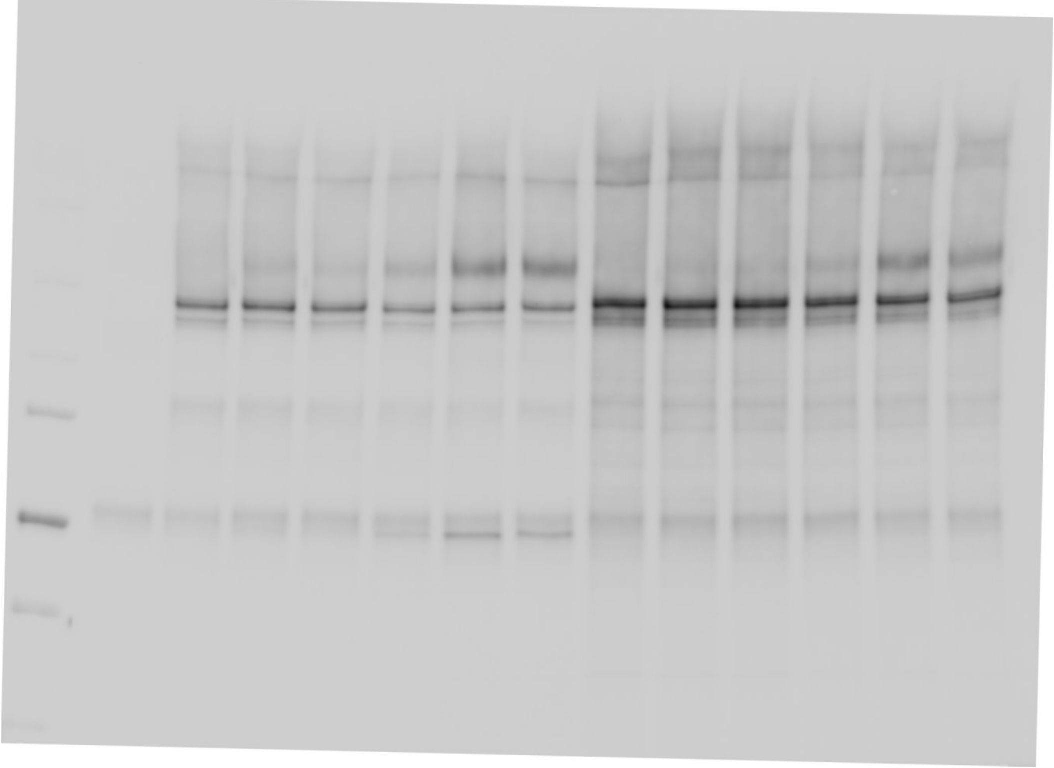

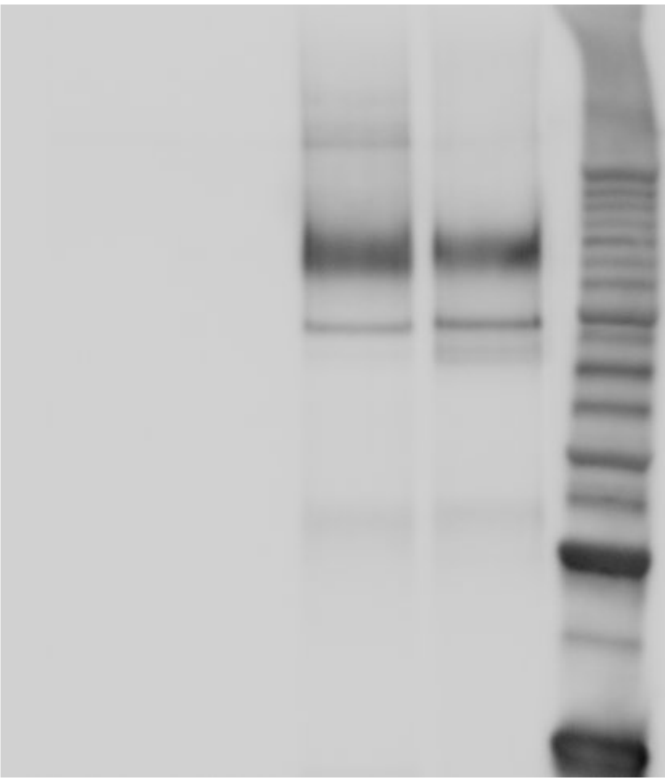

Fig 4A **IP:CFTR – WB Ub**

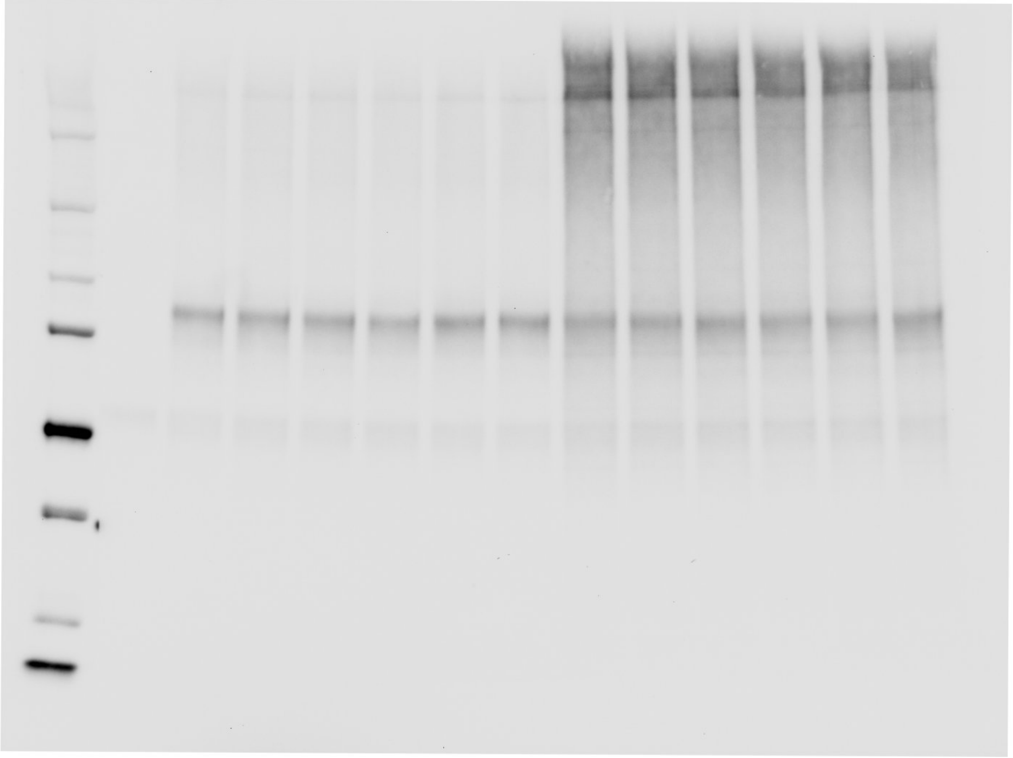

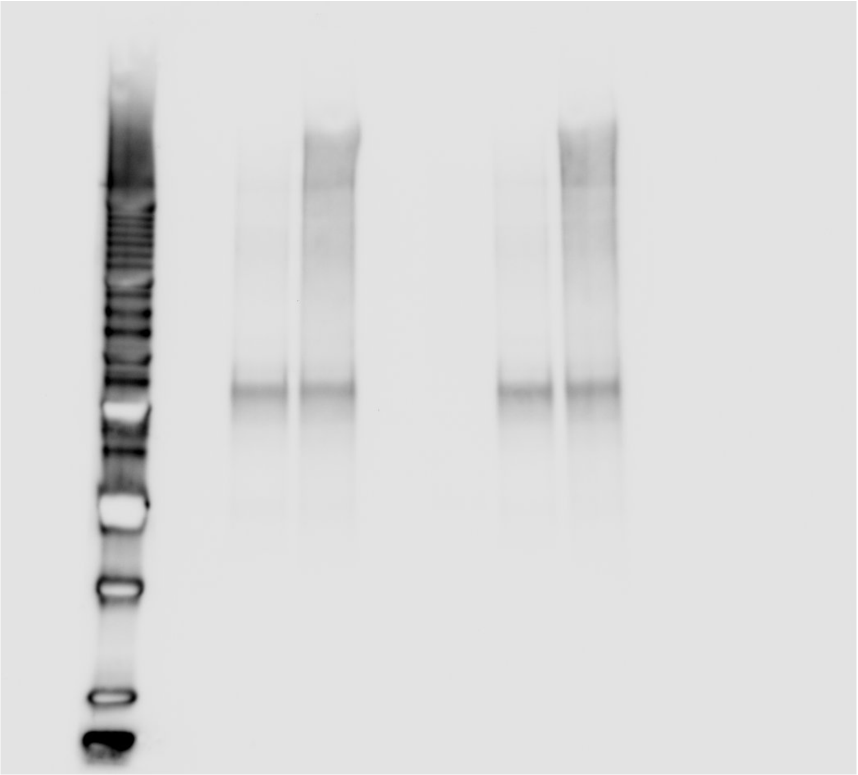

Fig 4A **Whole Lysates – WB CFTR/GAPDH**

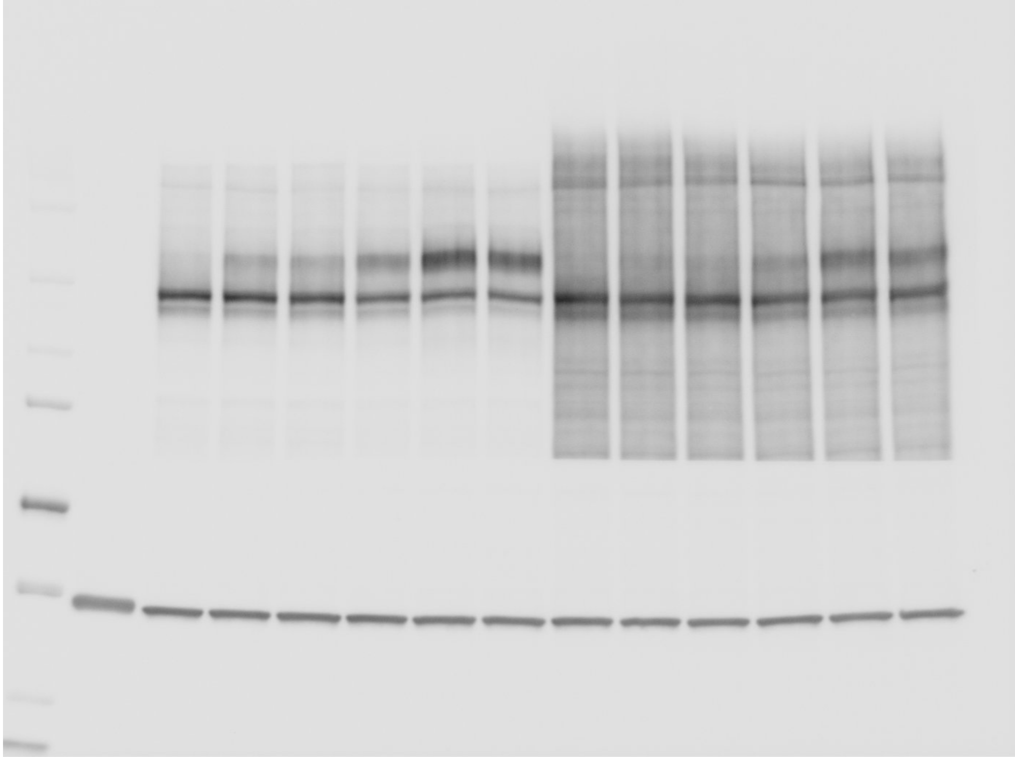

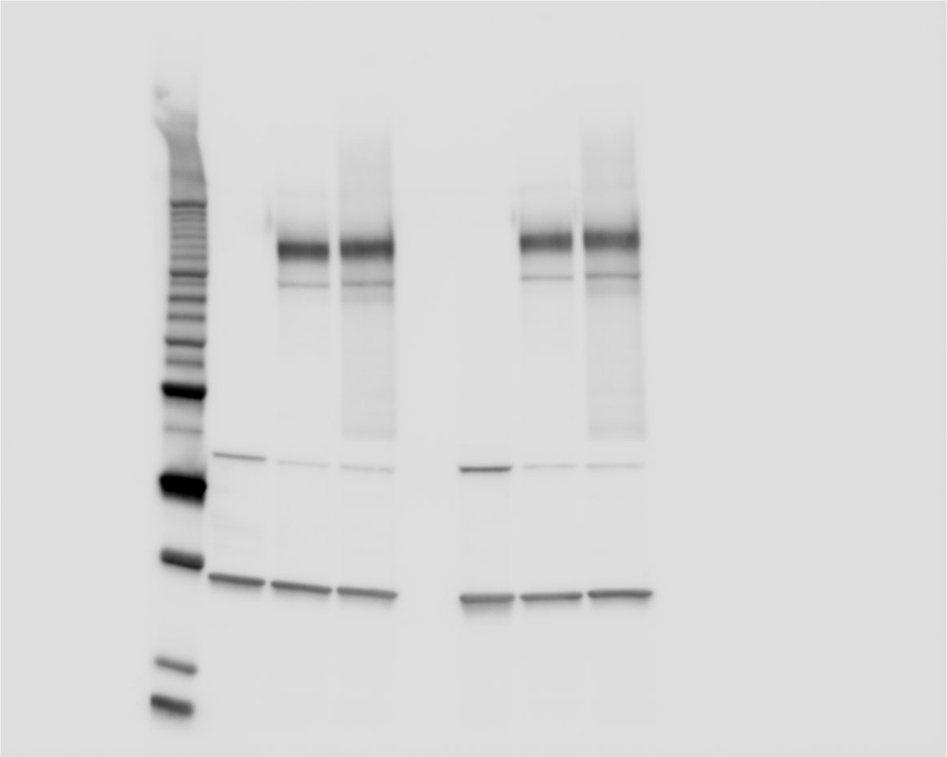

Fig 4A **Whole Lysates – WB Ub**

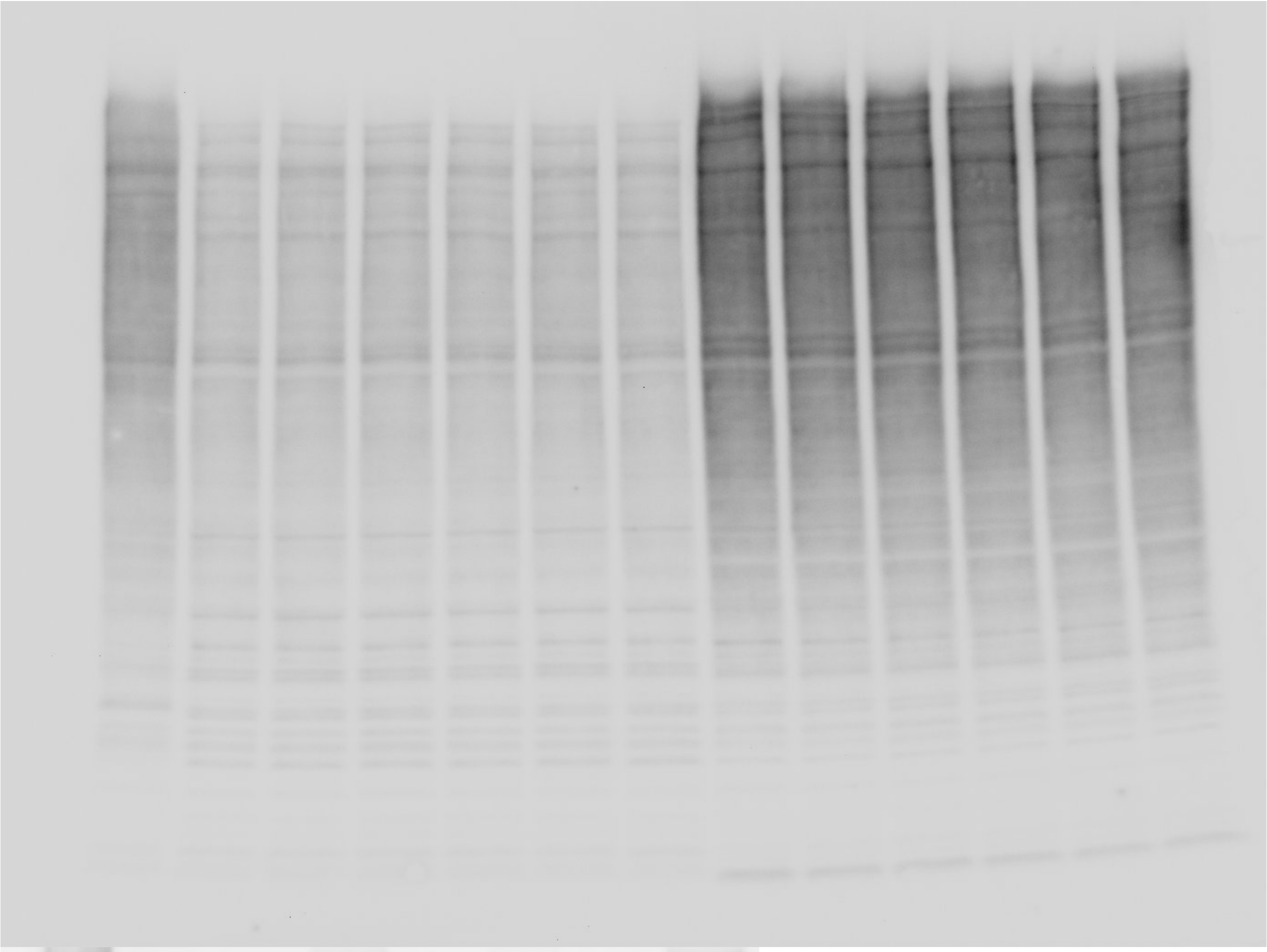

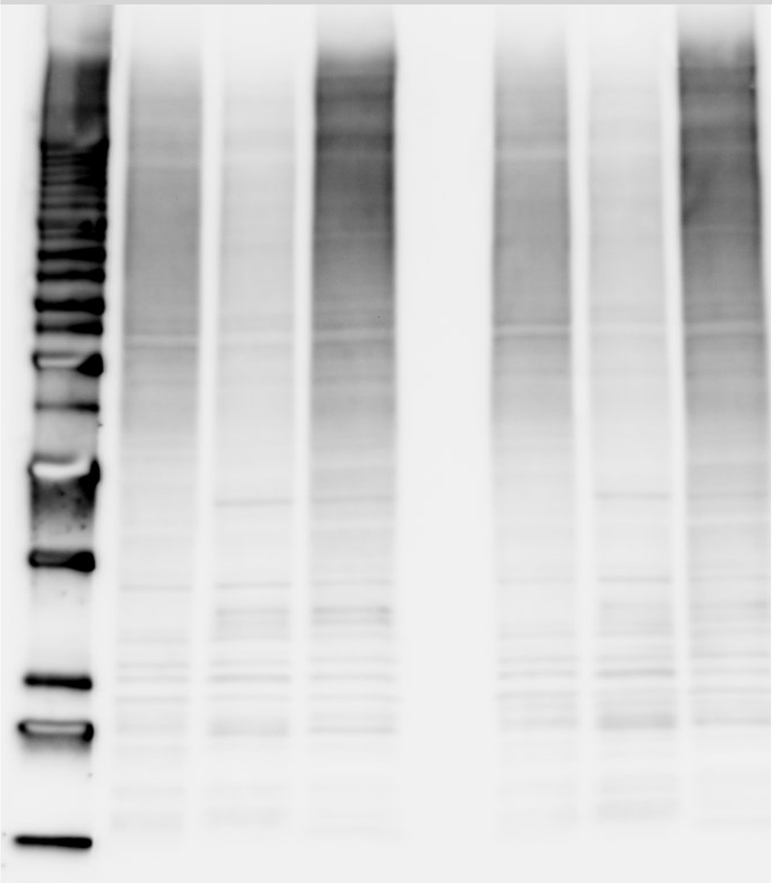

Fig 5A **CHX chase**

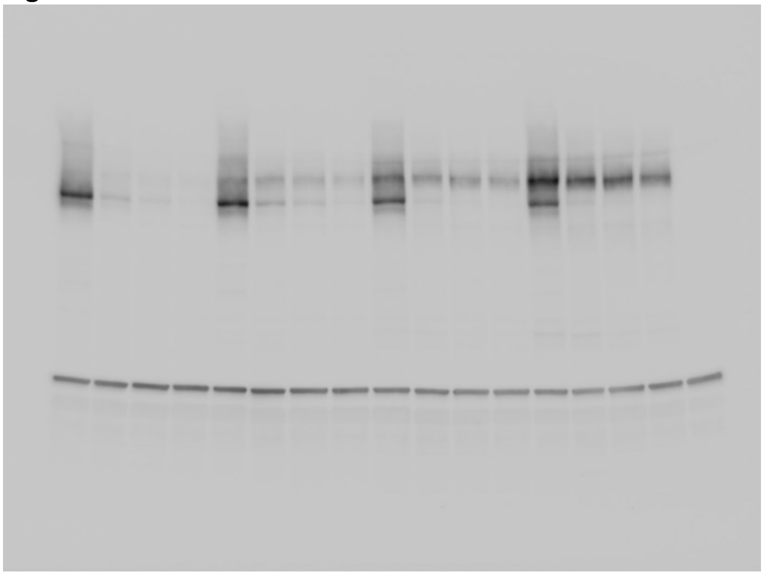

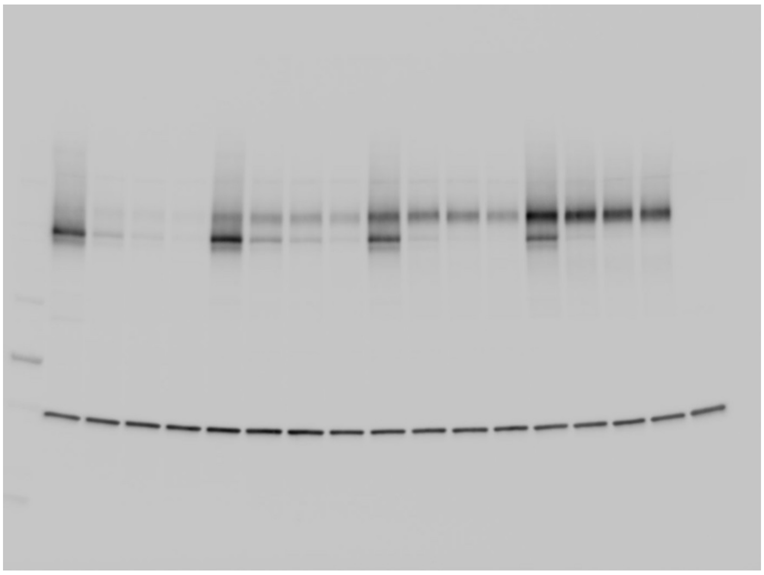

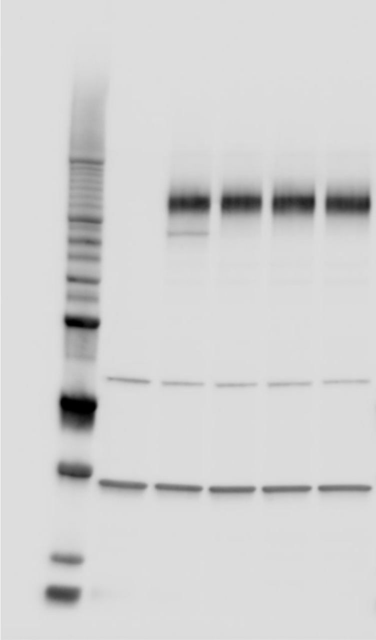

Fig 6A **Thermoaggregation assay**

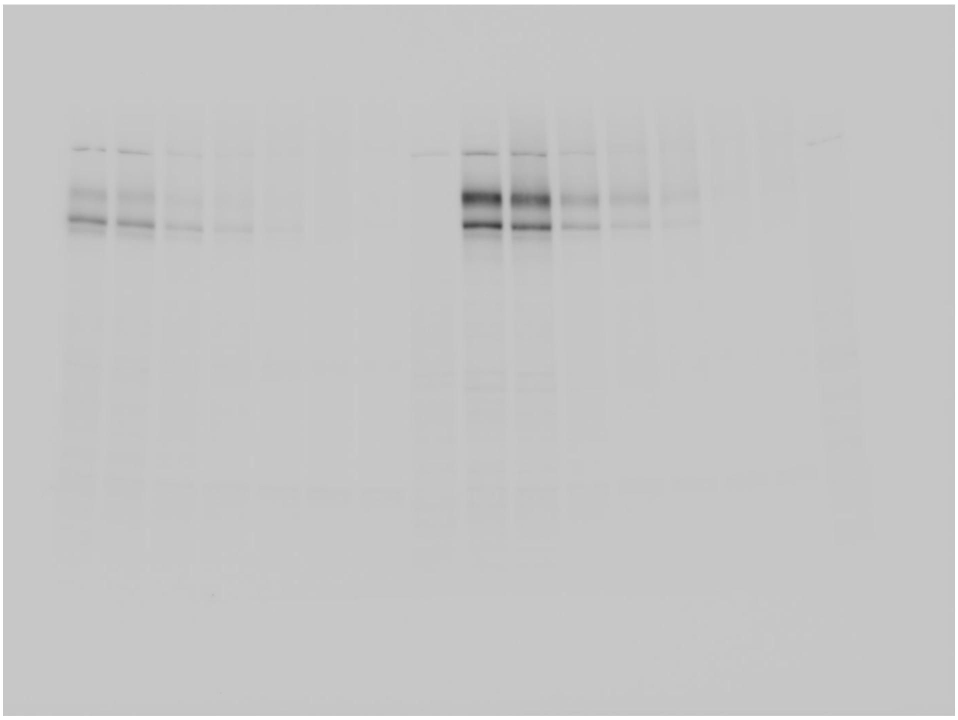

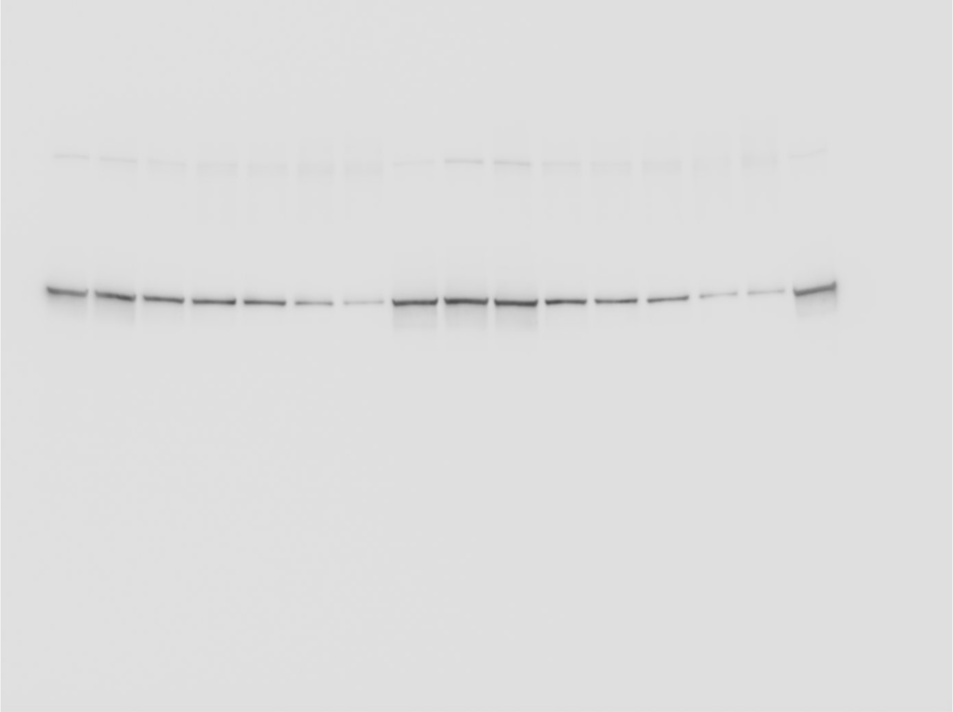

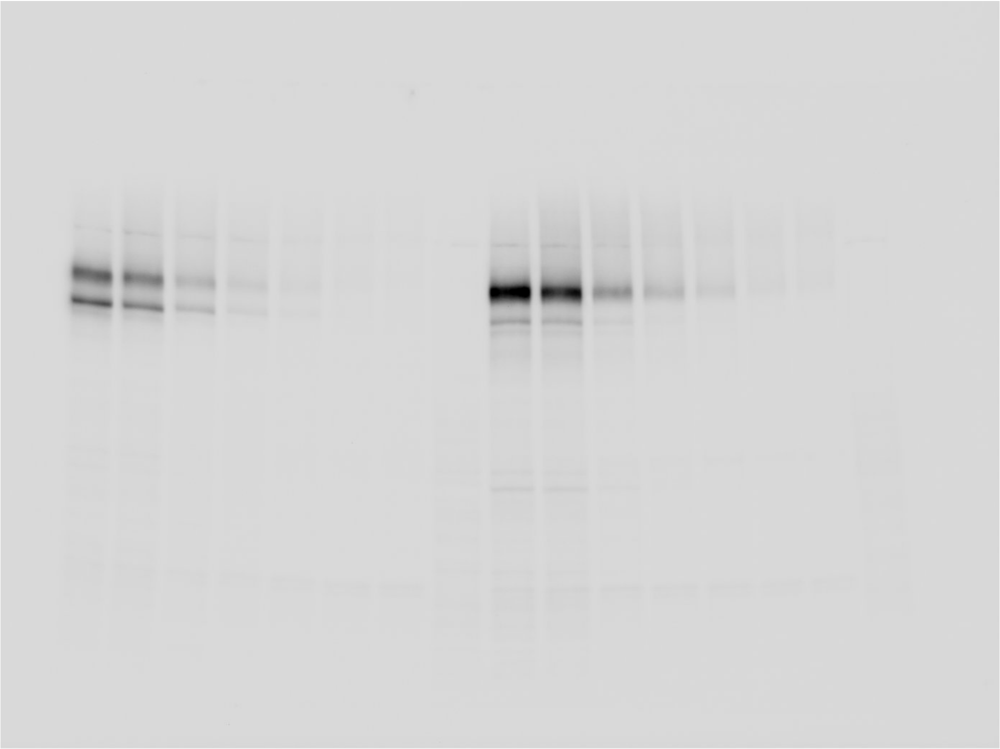

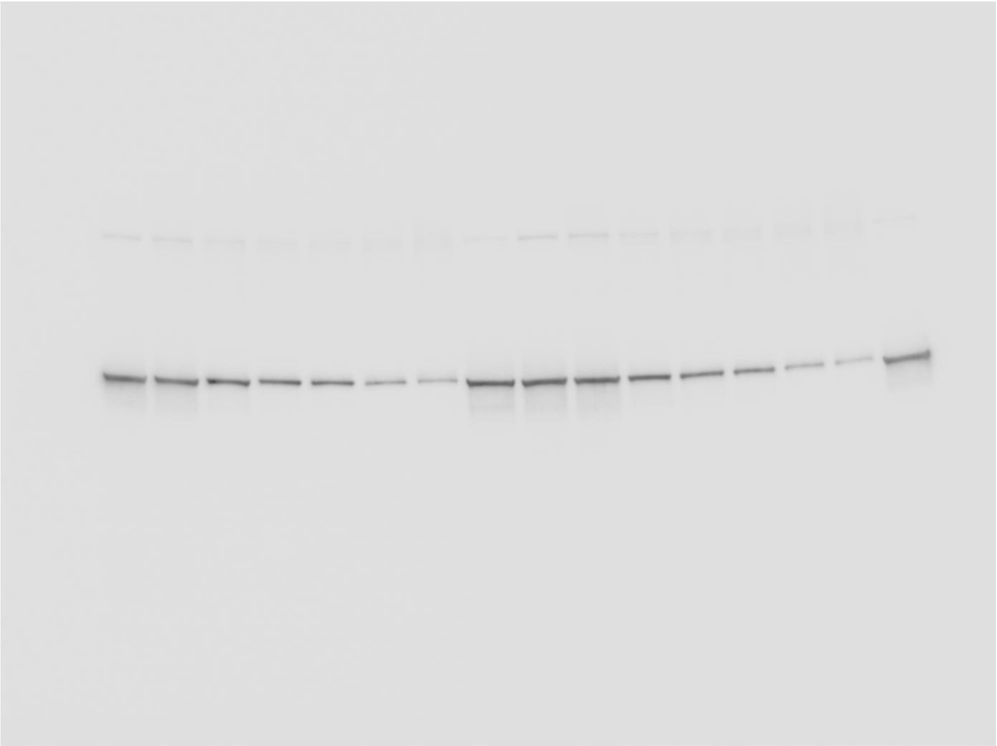

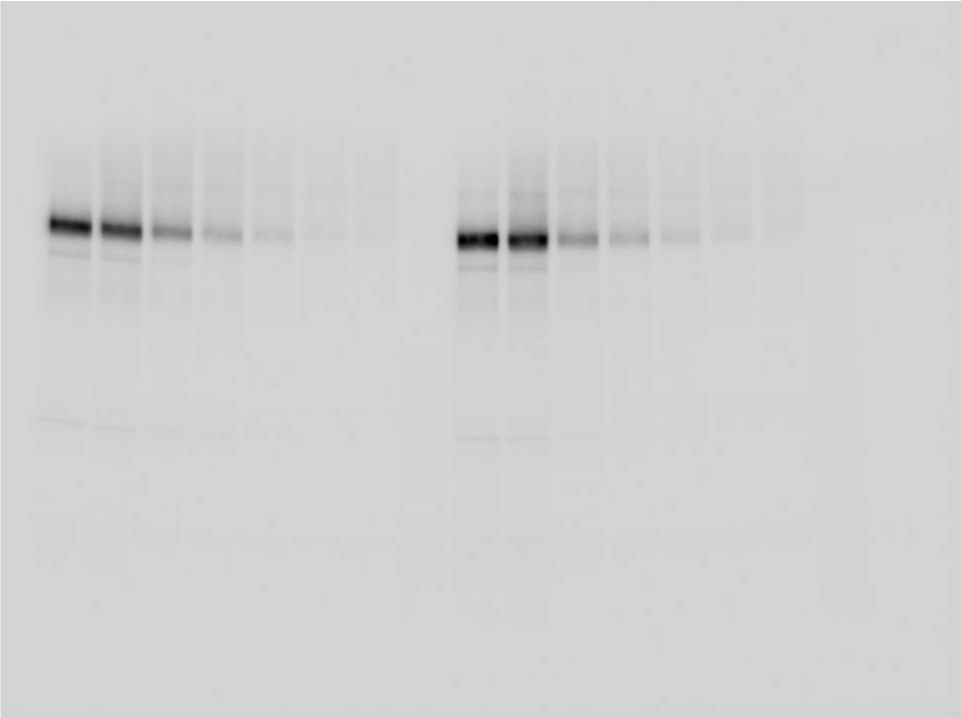

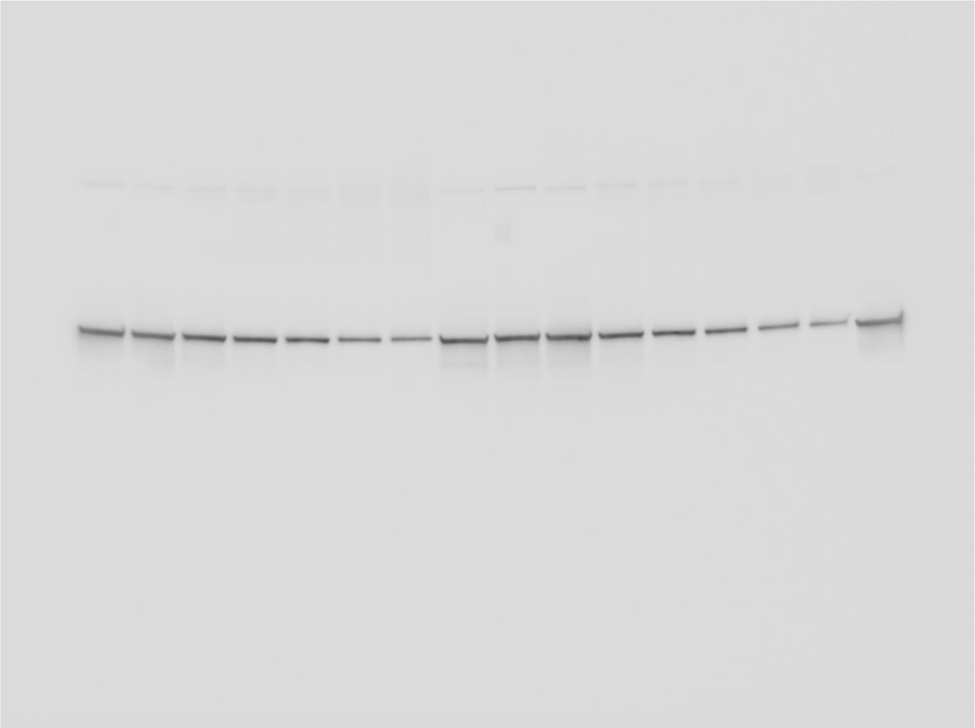

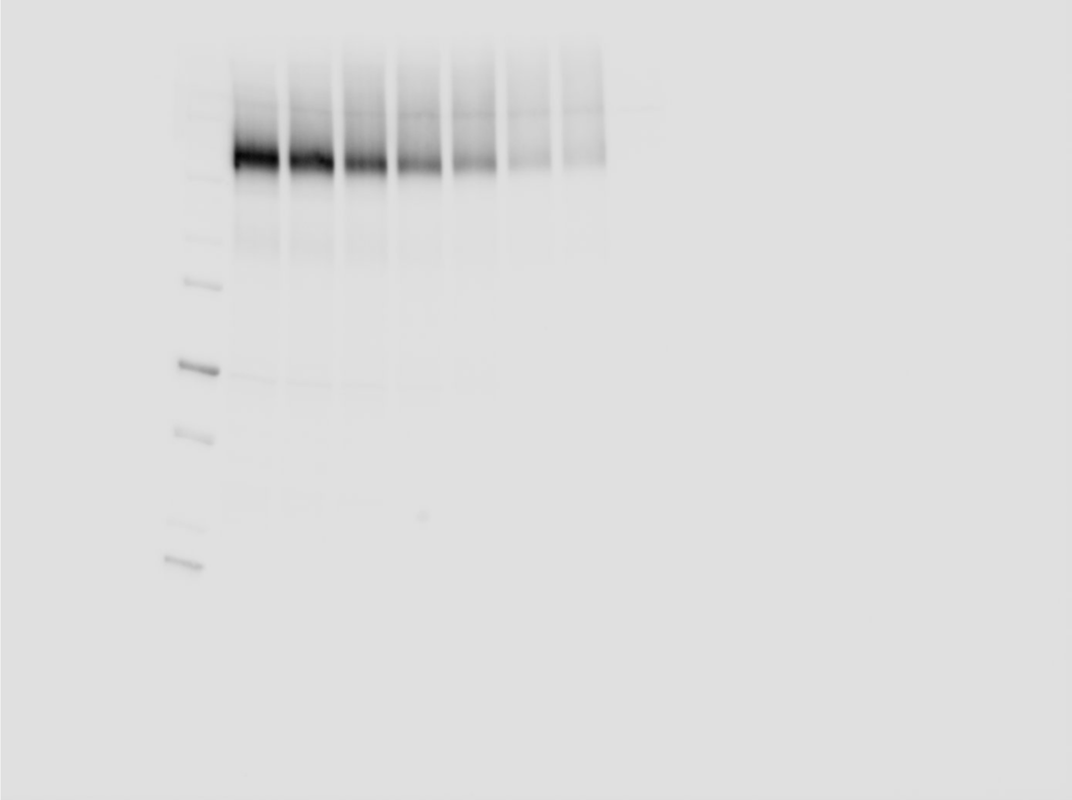

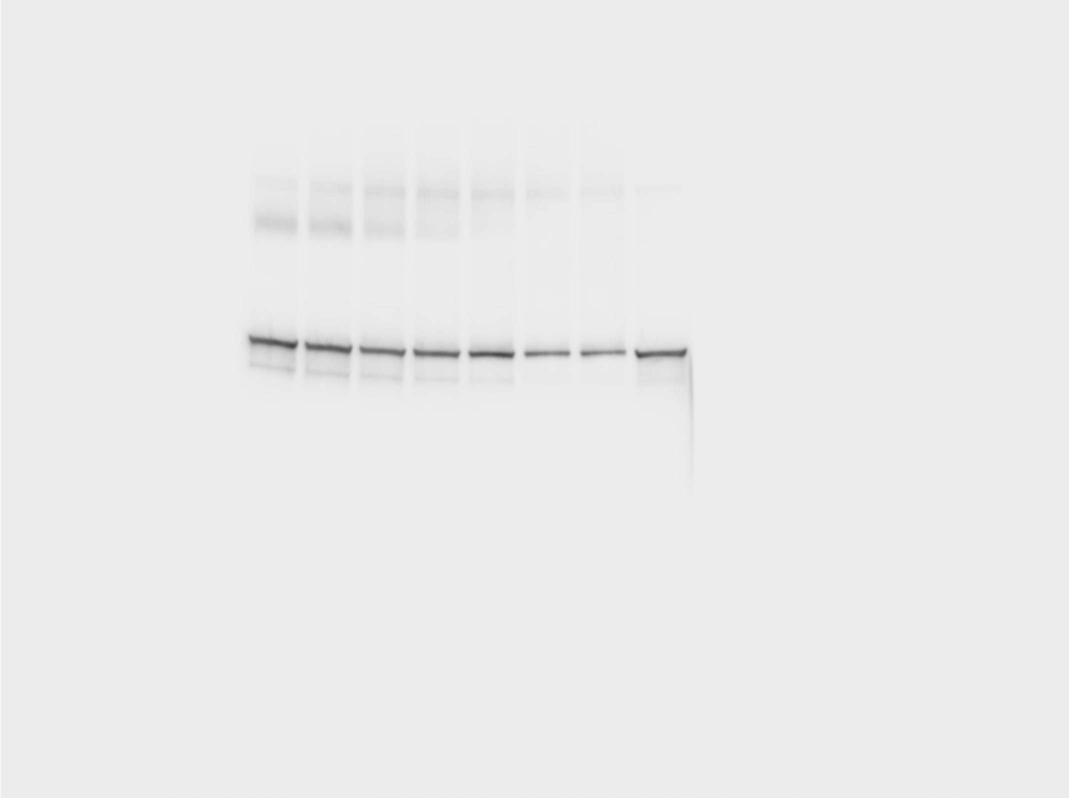

Supplement: Supplementary file 1 [file ijms-22-05262-s001.zip › uncropped.pdf]
