# Supplementary material for: Partial Rescue of F508del-CFTR Stability and Trafficking Defects by Double Corrector Treatment
Source: Int J Mol Sci. 2021 May 17;22(10):5262. doi: 10.3390/ijms22105262 (PMC8156943; doi:10.3390/ijms22105262)
Supplement: Supplementary file 1 [file ijms-22-05262-s001.zip › Suppl Info - CHEMISTRY.pdf]

Supporting Information

CHEMISTRY

Synthetic Materials and Methods

Compounds **racVX-445**, **VX-445** and its **(*R*)-enantiomer** were synthesized following a previously reported protocol [16]. Commercially available **VX-445** was purchased from MedchemExpress and used as reference compound for chiral HPLC analyses.

Solvents and reagents were obtained from commercial suppliers and were used without further purification. Automated column chromatography purifications were performed on Teledyne ISCO apparatus (CombiFlash® Rf) with pre-packed silica gel columns of different sizes (Redisep). NMR experiments were run on a Bruker Avance III 400 system (400.13 MHz for ^{1}H, and 100.62 MHz for ^{13}C), equipped with a BBI probe and Z-gradients and Bruker FT NMR Avance III 600 MHz spectrometer equipped with a 5 mm CryoProbeTM QCI ^{1}H/^{19}F–^{13}C/^{15}N–D quadruple resonance, a shielded z-gradient coil and the automatic sample changer SampleJet™ NMR system (600 MHz for ^{1}H, 151 MHz for ^{13}C and 565 MHz for ^{19}F). Chemical shifts for ^{1}H and ^{13}C spectra were recorded in parts per million using the residual non-deuterated solvent as the internal standard (for $CDCl_3$: 7.26 ppm, ^{1}H and 77.16 ppm, ^{13}C; for DMSO-d_6: 2.50 ppm, ^{1}H; 39.52 ppm, ^{13}C; for D_2O: 4.79 ppm, ^{1}H). The analyses by UPLC/MS were run on a Waters ACQUITY UPLC-MS system consisting of a Single Quadrupole Detector (SQD) mass spectrometer equipped with an Electrospray Ionization interface and a Photodiode Array Detector from Waters Inc. (Milford, MA, USA). The PDA range was 210-400 nm. Electrospray ionization in positive and negative mode was applied in the mass scan range 100-650 Da or 150-750 Da. The analyses were performed on an ACQUITY UPLC BEH C_{18} column (50 x 2.1 mm ID, particle size 1.7 µm) with a VanGuard BEH C_{18} pre-column (5 x 2.1 mm ID, particle size 1.7 µm) (LogD>1: *generic and apolar method*). The mobile phase was 10 mM NH_4OAc in H_2O at pH 5 adjusted with AcOH (A) and 10 mM NH_4OAc in MeCN-H_2O (95:5) at pH 5 (B) with 0.5 mL/min as flow rate. Different linear gradients were applied depending on LogD of the compounds: *generic method (LogD>1)*: 0-0.2 min: 5%B, 0.2-2.7 min: 5-95%B, 2.7-2.8 min: 95-100%B, 2.8-3.0 min: 100%B; *apolar method (LogD>1)*: 0-0.2 min: 50%B, 0.2-2.7 min: 50-100%B, 2.7-3.0 min: 100%B.

Compounds (*enant-1)*-**4a** and (*enant-2*)-**4b** were obtained by semi-preparative chiral HPLC on a Waters HPLC instrument consisting of a 1525 Binary HPLC Pump, 2998 Photodiode Array Detector and a Waters Fraction Collector III. The separation was performed on a Daicel ChiralCel ODH column (250 × 10 mm ID, particle size 5 µm) using Heptane/EtOH (95:5 v/v) as mobile phase (flow rate = 5 mL/min).

Determination of enantiomeric excess (%ee) for (*enant-1)*-**4a** and (*enant-2*)-**4b** was performed on a Waters Alliance HPLC instrument consisting of an e2695 Separation Module and a 2998 Photodiode Array Detector using a Daicel ChiralCel ODH column (250×4.6 mmID, particle size 5μm) and Heptane/EtOH (95:5 v/v) as mobile phase (flow rate = 1.0 mL/min).

Determination of enantiomeric excess (%ee) for **VX-445** and its **(*R*)-enantiomer** was performed on the HPLC system described above using a Daicel ChiralPak AD column (250 × 4.6 mm ID, particle size 10 μm) and Heptane/EtOH (95:5 v/v) as mobile phase (flow rate = 1.0 mL/min).

The absolute configuration of **VX-445** and its **(*R*)-enantiomer** synthesized in-house was determined by comparison of their specific rotation ($[\alpha]_D$) and chiral HPLC retention times (t_R) with a standard compound purchased from MedChemExpress.

Synthesis of intermediates (*enant-1*)-**4a** and (*enant-2*)-**4b**.

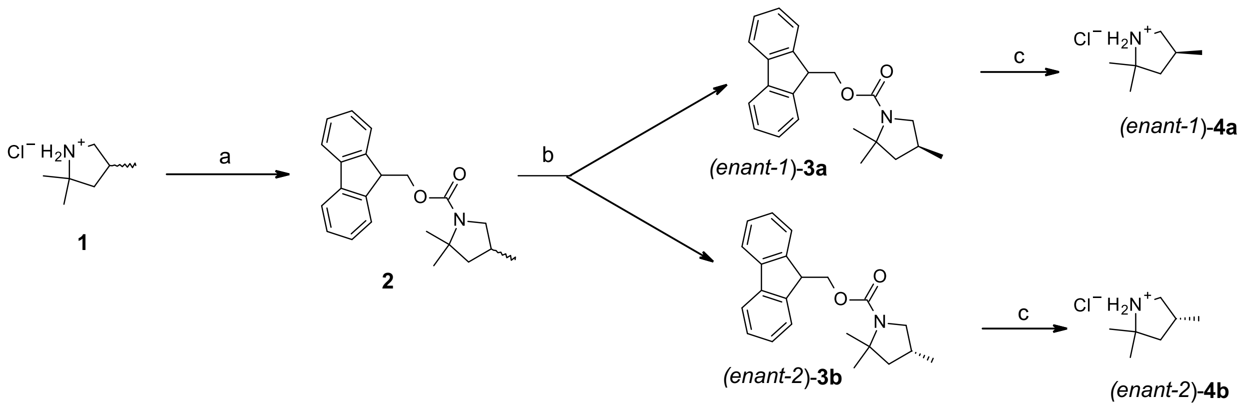

Scheme 1. *Reagents and conditions: (a)* 9-Fluorenylmethoxycarbonyl chloride, Na_2CO_3, H_2O, dioxane, room temperature; *(b)* chiral HPLC: column Daicel ChiralCel ODH, mobile phase: heptane/EtOH (95:5 v/v); *(c)* 2.0 M NaOH, dioxane, room temperature.

(*R/S*)-9*H*-Fluoren-9-ylmethyl-2,2,4-trimethylpyrrolidine-1-carboxylate (2). A solution of Na_2CO_3 (0.28 g, 2.61 mmol, 1.1 eq.) in H_2O (12 mL) was added to a mixture of commercially available racemic 2,2,4-trimethylpyrrolidine hydrochloride (**1**) (0.36 g, 2.37 mmol, 1.0 eq.) in dioxane (12 mL). The resulting mixture was cooled at 0 °C, 9-fluorenylmethoxycarbonyl chloride (Fmoc chloride) (0.68 g, 2.61 mmol, 1.1 eq.) was added and stirring was continued at room temperature for 24 h. The residue was taken in Et_2O (50 mL) and 0.5 M HCl solution (50 mL). The phase were partitioned and the water phase extracted with Et_2O (2 × 50 mL). The combined organic phase was washed with 0.5 M HCl solution (2 × 25 mL), brine (25 mL), dried over Na_2SO_4 and evaporated. Purification by flash chromatography (cyclohexane/EtOAc, 0%

to 15% EtOAc) afforded the pure title compound (0.62 g, 77%), as colorless sticky oil. ^{1}H NMR (400 MHz, $CDCl_3$): δ 7.81–7.71 (m, 2H), 7.65–7.55 (m, 2H), 7.44–7.27 (m, 4H), 4.66 (d, J = 4.5 Hz, 1H, *major rotamer*), 4.33 (d, J = 4.6 Hz, 1H, *minor rotamer*), 4.39–4.28 (m, 1H), 4.28–4.19 (m, 1H), 3.77–3.68 (m, 1H, *major rotamer*), 3.68–3.59 (m, 1H, *minor rotamer*), 2.94 (t, J = 10.5 Hz, 1H, *major rotamer*), 2.82 (t, J = 10.7 Hz, 1H, *minor rotamer*), 2.28 (ddq, J = 17.9, 12.2, 6.5 Hz, 1H, *major rotamer*), 2.11 (ddt, J = 17.9, 12.1, 6.5 Hz, 1H, *minor rotamer*), 1.91 (dd, J = 12.3, 6.2 Hz, 1H, *major rotamer*), 1.73 (dd, J = 12.4, 6.1 Hz, 1H *minor rotamer*), 1.49 (s, 3H, *major rotamer*), 1.47–1.42 (m, 1H, *major rotamer*), 1.35 (s, 3H, *major rotamer*), 1.33–1.28 (m, 1H, *minor rotamer*), 1.06 (d, J = 6.5 Hz, 3H, *major rotamer*), 0.94 (d, J = 6.4 Hz, 1H, *minor rotamer*), 0.84 (s, 3H, *minor rotamer*), 0.79 (s, 3H, *minor rotamer*). UPLC-MS: t_R = 2.19 min (apolar method). MS (ESI) m/z calcd. for $C_{22}H_{26}NO_2$ $[M+1]^+$: 336.2, found: 336.3.

9*H*-Fluoren-9-ylmethyl-2,2,4-trimethylpyrrolidine-1-carboxylate (*enant-1*)-3a and **(*enant-2*)-3b).** Compound **2** (0.32 g, 0.96 mmol) was purified under the semi-preparative HPLC conditions described above (please refer to *synthetic materials and methods section*). The fractions containing the pure enantiomers were evaporated *in vacuo* affording (*enant-1*)-**3a** (first eluted) and (*enant-2*)-**3b** (second eluted), as white solids. (*enant-1*)-**3a** (0.14 g, 41%): > 99.5% ee (λ = 254 nm), t_R 27.62 min; (*enant-2*)-**3b** (0.12 g, 36%): > 99.5% ee (λ = 254 nm), t_R 39.05 min. ^{1}H NMR and UPLC-MS of pure enantiomers were identical to those of the racemic mixture **2**.

(*R* or *S*)-2,2,4-Trimethylpyrrolidine hydrochloride (*enant-1*)-4a. Compound (*enant-1*)-**3a** (0.13 g, 0.39 mmol) was dissolved in dioxane (4.2 mL) and 2 M NaOH solution (581 µL, 1.16 mmol) was added. The resulting mixture was stirred overnight at room temperature. 1.0 M HCl solution (25 mL) was added to the reaction mixture and the water phase washed with Et_2O (3 × 25 mL). The organic phases were discarded and the water phase was evaporated affording the pure title compound (quant.), as white solid. The product contained inorganic salts (*i.e.,* NaCl) and was used in the next step without further purification. ^{1}H NMR (600 MHz, D_2O): δ 3.50 (dd, J = 11.8, 8.0 Hz, 1H), 2.91 (dd, J = 11.8, 9.5 Hz, 1H), 2.68–2.52 (m, 1H), 2.14 (dd, J = 13.2, 7.5 Hz, 1H), 1.54 (dd, J = 13.2, 10.4 Hz, 1H), 1.49 (s, 3H), 1.42 (s, 3H), 1.11 (d, J = 6.7 Hz, 3H).

(*S* or *R*)-2,2,4-Trimethylpyrrolidine hydrochloride (*enant-2*)-4b. Compound (*enant-2*)-**3b** (0.11 g, 0.331 mmol) was dissolved in dioxane (3.6 mL) and 2 M NaOH solution (496 µL, 0.993 mmol) was added. The resulting mixture was stirred overnight at room temperature. 1.0 M HCl solution (25 mL) was added to the reaction mixture and the water phase was washed with Et_2O (3 × 25 mL). The organic phases were discarded and the water phase was evaporated affording the pure title compound (quant.), as white solid. The product contained inorganic salts (*i.e.,* NaCl) and was used in the next step without further purification. ^{1}H NMR (600 MHz, D_2O): δ 3.50 (dd, J = 11.8, 8.0 Hz, 1H), 2.91 (dd, J = 11.8, 9.5 Hz, 1H), 2.68–2.52 (m, 1H),

2.14 (dd, J = 13.2, 7.5 Hz, 1H), 1.54 (dd, J = 13.2, 10.4 Hz, 1H), 1.49 (s, 3H), 1.42 (s, 3H), 1.11 (d, J = 6.7 Hz, 3H).

Synthesis of ***racVX-445***

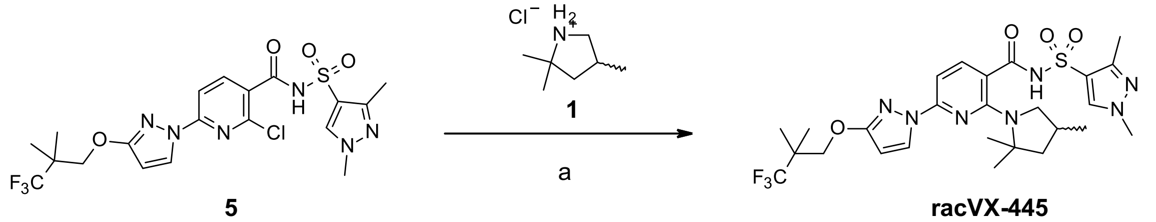

Scheme 2. *Reagents and conditions: (a)* K_2CO_3, DMSO, 130 °C, 20 h.

***(R/S)-N*-(1,3-dimethylpyrazol-4-yl)sulfonyl-6-[3-(3,3,3-trifluoro-2,2-dimethyl-propoxy)pyrazol-1-yl]-2-[2,2,4-trimethylpyrrolidin-1-yl]pyridine-3-carboxamide (racVX-445).** In a round-bottomed flask, under nitrogen and vigorous stirring, compound **5** [16] (0.06 g, 0.11 mmol, 1.0 eq.), commercially available 2,2,4-trimethylpyrrolidine hydrochloride (**1**) (0.05 g, 0.33 mmol, 3.0 eq.), and K_2CO_3 (0.091 g, 0.66 mmol, 6.0 eq.) were combined in anhydrous DMSO (0.5 mL) and stirred at 130 °C for 20 h. The reaction mixture was poured into H_2O (30 mL), the pH adjusted to 3-4 by adding 2 M HCl and extraction with Et_2O (3 × 25 mL) was performed. The combined organic phase was washed with brine (2 × 20 mL), dried over Na_2SO_4 and evaporated. Purification by flash chromatography (cyclohexane/EtOAc, gradient 0%-40% EtOAc) afforded an impure product. A second flash chromatography (CH_2Cl_2/MeOH 8:2) was performed giving a pure product, which was freeze-dried to afford the title compound (0.035 g, 53%), as white solid. ^{1}H NMR (400 MHz, DMSO-d_6): δ 12.33 (s, 1H), 8.34 (s, 1H), 8.21 (d, J = 2.7 Hz, 1H), 7.73 (d, J = 8.2 Hz, 1H), 6.93 (d, J = 8.2 Hz, 1H), 6.16 (d, J = 2.7 Hz, 1H), 4.23 (s, 2H), 3.80 (s, 3H), 2.57 (t, J = 10.4 Hz, 1H), 2.48–2.39 (m, 1H), 2.32 (s, 3H), 2.25–2.08 (m, 1H), 1.87 (dd, J = 12.0, 5.6 Hz, 1H), 1.56 (s, 3H), 1.53 (s, 3H), 1.42 (t, J = 12.1 Hz, 1H), 1.23 (s, 6H), 0.81 (d, J = 6.3 Hz, 3H). ^{13}C NMR (101 MHz, DMSO-d_6): δ 165.2, 164.0, 152.3, 148.9, 146.7, 141.5, 136.4, 128.6 (q, J = 285.4 Hz), 128.4, 117.3, 111.2, 96.2, 95.3, 70.9, 64.0, 57.7, 50.8, 40.8 (q, J = 24.3 Hz), 38.7, 29.7, 26.4, 25.0, 18.1, 16.4, 12.0. UPLC-MS: t_R = 2.51 min (generic method). MS (ESI) m/z calcd for $C_{26}H_{35}F_3N_7O_4S$ $[M+1]^+$: 598.2, found: 598.4. Chiral HPLC: *enant-1* and *enant-2*, t_R = 17.28 and 25.50 min, respectively (see Chiral analyses).

Synthesis of ***VX-445***

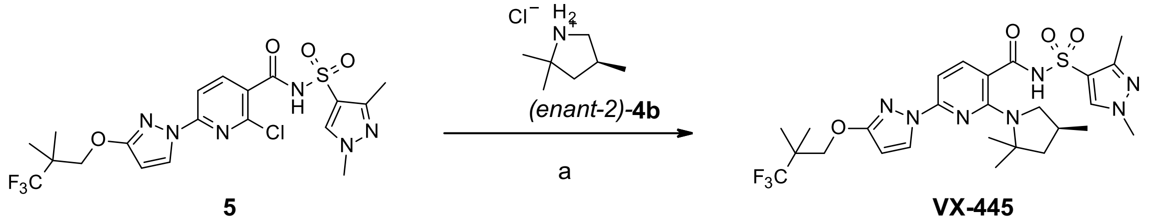

Scheme 3. *Reagents and conditions: (a)* K_2CO_3, DMSO, 130 °C.

***N*-(1,3-dimethylpyrazol-4-yl)sulfonyl-6-[3-(3,3,3-trifluoro-2,2-dimethyl-propoxy)pyrazol-1-yl]-2-[(*S*)-2,2,4-trimethylpyrrolidin-1-yl]pyridine-3-carboxamide (VX-445). VX-445** was synthesized from compound **5** (0.058 g, 0.11 mmol, 1.0 eq.) and (*enant-2*)-**4b** (0.33 theoretical mmol, 3.0 eq.), as previously reported [16] $[\alpha]_D^{27}$ – 45.682 (c 0.199, $CHCl_3$). Chiral HPLC: > 99.4% ee (λ = 275 nm), t_R = 25.24 min (see Chiral analyses).

Synthesis of ***(R)-enantiomer***

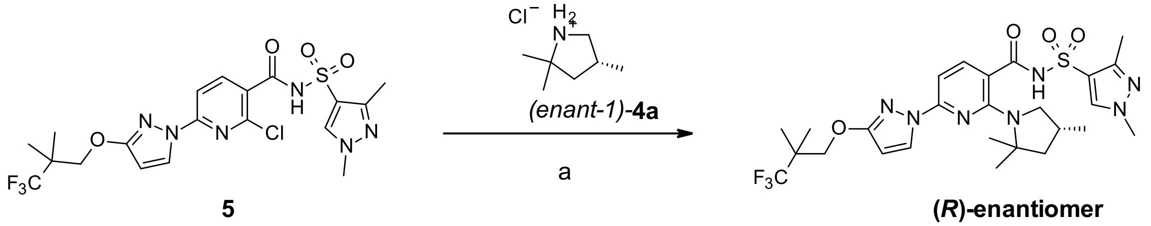

Scheme 4. *Reagents and conditions: (a)* K_2CO_3, DMSO, 130 °C.

***N*-(1,3-dimethylpyrazol-4-yl)sulfonyl-6-[3-(3,3,3-trifluoro-2,2-dimethyl-propoxy)pyrazol-1-yl]-2-[(*R*)-2,2,4-trimethylpyrrolidin-1-yl]pyridine-3-carboxamide [(*R*)-enantiomer]. (*R*)-enantiomer** of racVX-445 was synthesized from compound **5** (0.058 g, 0.11 mmol, 1.0 eq.) and (*enant-1*)-**4a** (0.33 theoretical mmol, 3.0 eq.), as reported for the synthesis of racVX-445. $[\alpha]_D^{27}$ + 58.292 (c 0.212, $CHCl_3$). Chiral HPLC: > 99.4% ee (λ = 275 nm), t_R = 16.54 min (see Chiral analyses).

*Chiral HPLC analyses of **racVX-445**, **(R)-enantiomer**, **VX-445** (in-house synthesis), **VX-445** (purchased) and **VX-445** (co-injection).*

(R/S)-N-(1,3-dimethylpyrazol-4-yl)sulfonyl-6-[3-(3,3,3-trifluoro-2,2-dimethyl-propoxy)pyrazol-1-yl]-2-[2,2,4-trimethylpyrrolidin-1-yl]pyridine-3-carboxamide **(racVX-445)** from in-house synthesis.

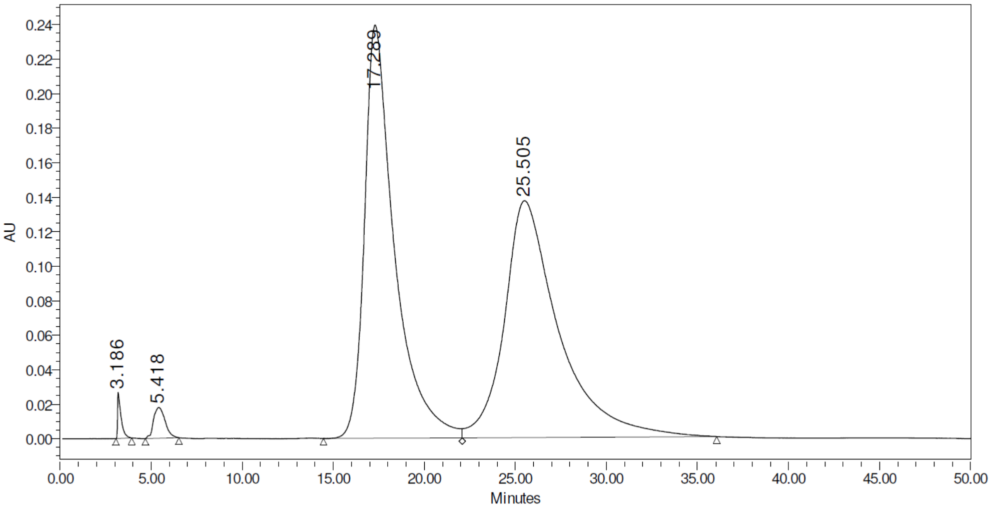

	RT	Area	% Area	Height
1	3.186	352977	0.62	26605
2	5.418	782970	1.37	17819
3	17.289	27454194	48.08	239410
4	25.505	28512429	49.93	137245

*N-(1,3-dimethylpyrazol-4-yl)sulfonyl-6-[3-(3,3,3-trifluoro-2,2-dimethyl-propoxy)pyrazol-1-yl]-2-[(R)-2,2,4-trimethylpyrrolidin-1-yl]pyridine-3-carboxamide [**(R)-enantiomer**]* from in-house synthesis.

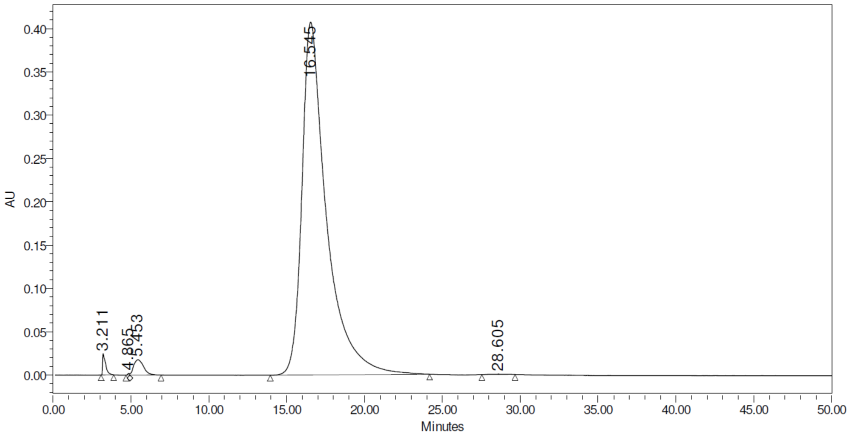

	RT	Area	% Area	Height
1	3.211	333950	0.73	24567
2	4.865	17168	0.04	1918
3	5.453	782686	1.71	17785
4	16.545	44669158	97.46	407298
5	28.605	31461	0.07	431

$[\alpha]_D^{27}$ + 58.292 (c 0.212, $CHCl_3$). Chiral HPLC: > 99.4% ee (λ = 275 nm), t_R = 16.54 min

*N-(1,3-dimethylpyrazol-4-yl)sulfonyl-6-[3-(3,3,3-trifluoro-2,2-dimethyl-propoxy)pyrazol-1-yl]-2-[(S)-2,2,4-trimethylpyrrolidin-1-yl]pyridine-3-carboxamide (**VX-445**)* from in-house synthesis.

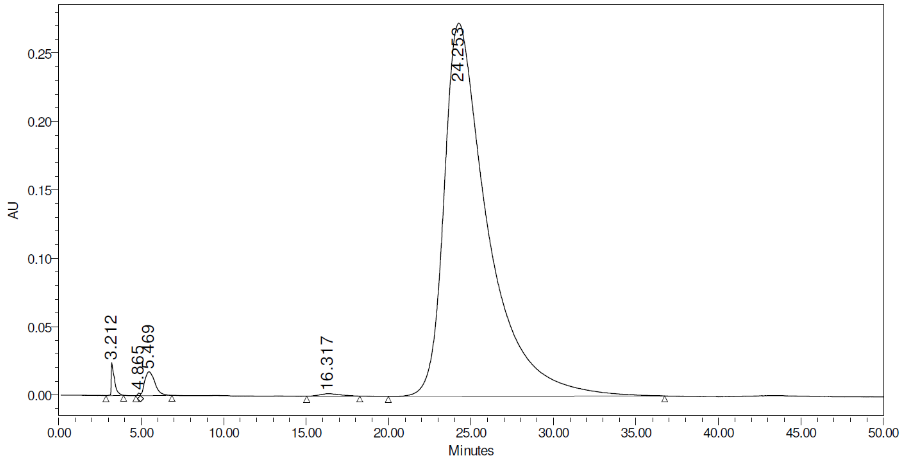

	RT	Area	% Area	Height
1	3.212	328516	0.64	23979
2	4.865	18528	0.04	1791
3	5.469	739628	1.43	17480
4	16.317	143020	0.28	1730
5	24.253	50350482	97.62	272851

$[\alpha]_D^{27}$ – 45.682 (c 0.199, $CHCl_3$). Chiral HPLC: > 99.4% ee (λ = 275 nm), t_R = 25.24 min

*N-(1,3-dimethylpyrazol-4-yl)sulfonyl-6-[3-(3,3,3-trifluoro-2,2-dimethyl-propoxy)pyrazol-1-yl]-2-[(S)-2,2,4-trimethylpyrrolidin-1-yl]pyridine-3-carboxamide (**VX-445**)* purchased from MedChemExpress.

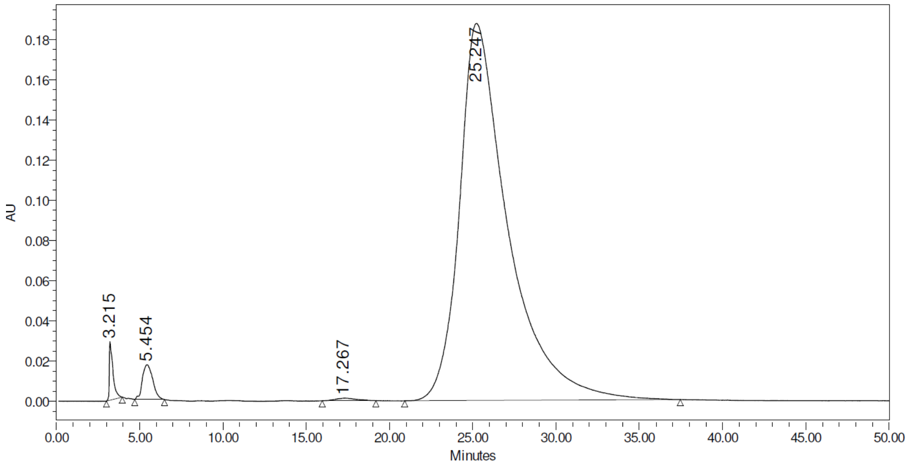

	RT	Area	% Area	Height
1	3.215	445703	1.13	29131
2	5.454	752981	1.91	17382
3	17.267	113556	0.29	1289
4	25.247	38083443	96.67	187786

$[\alpha]_D^{27}$ – 48.616 (c 0.222, $CHCl_3$). Chiral HPLC: > 99.4% ee (λ = 275 nm), t_R = 25.24 min

Co-injection of in-house synthesized and purchased ***VX-445***

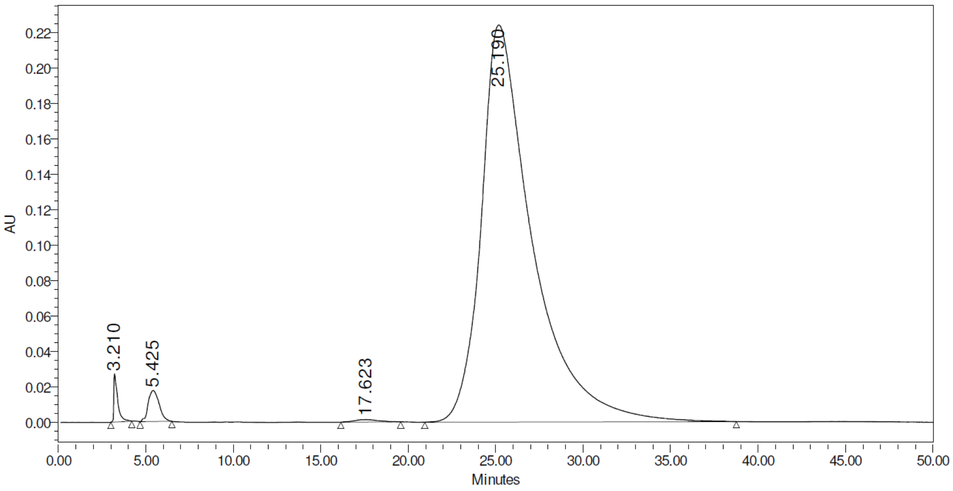

	RT	Area	% Area	Height
1	3.210	421065	0.90	27330
2	5.425	758307	1.62	17514
3	17.623	133400	0.29	1394
4	25.190	45477634	97.19	223973

Chiral HPLC: > 99.6% ee (λ = 275 nm), t_R = 25.19 min
